# Classifying Intracortical Brain-Machine Interface Signal Disruptions Based on System Performance and Applicable Compensatory Strategies: A Review

**DOI:** 10.3389/fnbot.2020.558987

**Published:** 2020-10-09

**Authors:** Collin F. Dunlap, Samuel C. Colachis, Eric C. Meyers, Marcia A. Bockbrader, David A. Friedenberg

**Affiliations:** ^1^Department of Biomedical Engineering, The Ohio State University, Columbus, OH, United States; ^2^Medical Devices and Neuromodulation, Battelle Memorial Institute, Columbus, OH, United States; ^3^Department of Physical Medicine and Rehabilitation, The Ohio State University, Columbus, OH, United States; ^4^Advanced Analytics and Health Research, Battelle Memorial Institute, Columbus, OH, United States

**Keywords:** microelectrode failure, biocompatibility, recording disruptions, neuroprosthetics, brain-computer interface, intracortical electrode array, signal quality

## Abstract

Brain-machine interfaces (BMIs) record and translate neural activity into a control signal for assistive or other devices. Intracortical microelectrode arrays (MEAs) enable high degree-of-freedom BMI control for complex tasks by providing fine-resolution neural recording. However, chronically implanted MEAs are subject to a dynamic *in vivo* environment where transient or systematic disruptions can interfere with neural recording and degrade BMI performance. Typically, neural implant failure modes have been categorized as biological, material, or mechanical. While this categorization provides insight into a disruption's causal etiology, it is less helpful for understanding degree of impact on BMI function or possible strategies for compensation. Therefore, we propose a complementary classification framework for intracortical recording disruptions that is based on duration of impact on BMI performance and requirement for and responsiveness to interventions: (1) *Transient disruptions* interfere with recordings on the time scale of minutes to hours and can resolve spontaneously; (2) *Reversible disruptions* cause persistent interference in recordings but the root cause can be remedied by an appropriate intervention; (3) *Irreversible compensable disruptions* cause persistent or progressive decline in signal quality, but their effects on BMI performance can be mitigated algorithmically; and (4) *Irreversible non-compensable disruptions* cause permanent signal loss that is not amenable to remediation or compensation. This conceptualization of intracortical BMI disruption types is useful for highlighting specific areas for potential hardware improvements and also identifying opportunities for algorithmic interventions. We review recording disruptions that have been reported for MEAs and demonstrate how biological, material, and mechanical mechanisms of disruption can be further categorized according to their impact on signal characteristics. Then we discuss potential compensatory protocols for each of the proposed disruption classes. Specifically, transient disruptions may be minimized by using robust neural decoder features, data augmentation methods, adaptive machine learning models, and specialized signal referencing techniques. Statistical Process Control methods can identify reparable disruptions for rapid intervention. *In-vivo* diagnostics such as impedance spectroscopy can inform neural feature selection and decoding models to compensate for irreversible disruptions. Additional compensatory strategies for irreversible disruptions include information salvage techniques, data augmentation during decoder training, and adaptive decoding methods to down-weight damaged channels.

## Introduction

**Figure 1:**
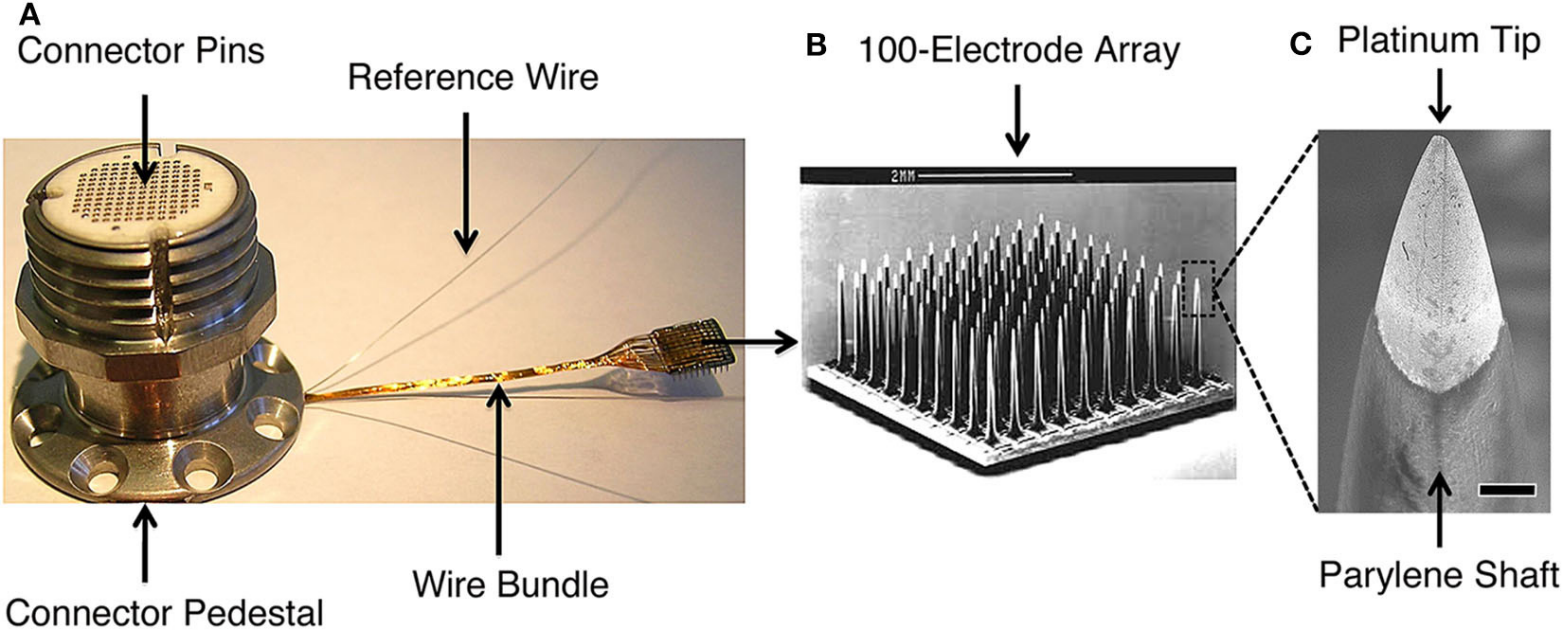
The Blackrock Microsystems (Salt Lake, UT) silicon-based, Utah microelectrode array used in high performance intracortical brain-machine interface trials (e.g., Wodlinger et al., 2015; Pandarinath et al., 2017; Bockbrader et al., 2019). **(A)** Skull-mounted titanium pedestal with the wire bundle connected to a Utah MEA. The pedestal connector pins interface with an analog or digital headstage that transmits signals to the data acquisition system. **(B)** Scanning electron microscopy (SEM) image of the 10x10 array (scale bar is 2 mm). **(C)** SEM of platinum-coated silicon tip and parylene-coated silicon shaft of a MEA electrode (scale bar is 10 μm). MEA signals may be disrupted, e.g., by biologic tissue reactions around the electrode tips, deterioration of electrode materials, or mechanical connection failures. Detection methods can leverage the characteristic manner in which disruptions affect recorded signals, allowing for targeted interventions to restore signal quality and BMI function. Figure reprinted with permission from Barrese et al. (2016). © IOP Publishing. All rights reserved.

Brain machine interface systems (BMIs) have been proposed as assistive devices to restore, replace, or augment lost motor function to people with paralysis (Hochberg et al., 2006; Collinger et al., 2013b; Aflalo et al., 2015; Bouton et al., 2016; Ajiboye et al., 2017). These neural interface systems record and interpret brain signals, enabling control of an effector device through modulation of neural activity. Non-invasive neural recording techniques including electroencephalography (EEG), functional near-infrared spectroscopy (fNIRS), and functional magnetic resonance imaging (fMRI) can function as sensors for BMIs (Hochberg and Donoghue, 2006; Nicolas-Alonso and Gomez-Gil, 2012). However, these systems lack the spatiotemporal resolution or information transfer capacity required for accurate and intuitive control of high degree-of-freedom (DOF) effectors (Hochberg and Donoghue, 2006; Lebedev and Nicolelis, 2006; Klaes, 2018). In contrast, invasive recording systems utilizing electrocorticography (ECoG) grids or microelectrode arrays (MEAs) provide a richer source of neural information. Penetrating cortical MEAs including microwire arrays (Nicolelis et al., 2003; Krüger et al., 2010; Schwarz et al., 2014; Obaid et al., 2020), Michigan-style arrays (Wise, 2005), and the Utah array (Figure 1; Campbell et al., 1991; Leber et al., 2019) acquire neural activity with unparalleled signal resolution. MEAs can record single- and multi-unit neural activity correlated with the kinetics (Fagg et al., 2009) and kinematics (Vargas-Irwin et al., 2010; Aggarwal et al., 2013) of the arm during reaching and grasp (Downey et al., 2018b). Such fine-resolution recordings have enabled intuitive control of high DOF robotic arms (Wodlinger et al., 2015) and functional electrical stimulation (FES) systems (Colachis et al., 2018) by people with quadriplegia. MEAs can also provide the necessary somatosensory feedback-control to enhance grip performance of high DOF robotic arms (Flesher et al., 2017) and advanced FES orthotics (Ganzer et al., 2020). This review focuses on recording disruptions that affect MEA BMI performance and limit their use.

Although their performance capabilities are impressive, a significant barrier to the widespread adoption of intracortical neural interfaces as assistive devices is the limited lifetime of the recording array. The most extensive retrospective study of intracortical MEA failure modes to date showed that a majority of devices failed within a year of implantation in non-human primates (NHPs) (Barrese et al., 2013). Even in the absence of acute failures, analyses of state-of-the-art MEAs predict they have less than a decade of useful life due to persistent decline in recording quality over time (Barrese et al., 2013). Less is known about the longevity of MEAs in humans due to the limited number of studies utilizing chronic implants. The few clinical trials that have investigated the functionality of intracortical BMIs beyond 4 years post-implant have reported sustained usability (Hochberg et al., 2012; Bockbrader, 2019; Bockbrader et al., 2019; Hughes et al., 2020), while most other clinical studies had planned MEA explantation dates that occurred prior to MEA malfunction (Bullard et al., 2020). While the useable life of MEAs for BMI control is likely longer in humans than has been reported in NHP studies, chronic declines in signal quality are evident in human trials (Perge et al., 2014; Zhang et al., 2018; Hughes et al., 2020). These declines are sometimes persistent, progressive, and irreversible because the underlying disruptions affect the tissue-electrode interface (e.g., glial scarring and meningeal encapsulation), or implanted hardware (e.g., electrode insulation deterioration). Such signal disruptions require neurosurgical intervention for hardware exchange to completely resolve. Device replacement to improve performance or evaluate the hardware for damage can be both prohibitively costly and risky, involving potential for cortical tissue injury during explant and reimplant as well as risk for infection, hemorrhage, and adverse reaction to anesthesia. Identifying chronic signal disruption types that may be amenable to non-surgical remediation could potentially extend the longevity and enhance the attractiveness of intracortical BMIs as assistive neurotechnology.

Another challenge affecting practical usability of intracortical neural interfaces is dynamic neural signal drift and other transient disruptions. For example, the presence of an object in a neuroprosthetic reach and grasp task may transiently affect neural population firing rates and complicate decoding of intended grip states (Downey et al., 2017). Additionally, micromovements of the MEA and cognitive fatigue can impact how neural features are represented across channels over time. A common technique used with humans to mitigate these signal instabilities is to train intracortical BMI algorithms *de novo* on a daily basis (Hochberg et al., 2012; Collinger et al., 2013b; Bouton et al., 2016; Ajiboye et al., 2017). However, transient disruptions can decrease BMI performance to chance levels in as little as 30 min (Perge et al., 2013). This effectively renders the interface useless until the disruption is resolved, or the decoder is recalibrated. Recalibration prolongs set up time, a characteristic that candidate BMI users rate as very important to minimize (Collinger et al., 2013a). Other transient disruptions, e.g., FES stimulation artifact (Bouton et al., 2016), introduce noise into the recording that must be removed to avoid temporary loss of control when operating a physical effector like a grip orthotic. Therefore, recognizing and accounting for transient signal instabilities are important ways to improve convenience, safety, and eventual adoption of BMI systems.

Consequently, detecting and mitigating MEA signal disruptions on both chronic and acute time scales are important, open challenges for the field. As a first step, researchers have identified and classified common MEA failures (Barrese et al., 2013, 2016; Prasad et al., 2014; Wellman et al., 2018). Typically, the root causes of these failures have been sorted into three main categories: biological, material, or mechanical. This organization is convenient for grouping failures with similar underlying causes and helps establish design criteria for next-generation neural implants. For example, mechanical design considerations include that electrodes should be strong enough to withstand the physical forces during cortical insertion, but also sufficiently compliant to minimize micromotion-induced strain on surrounding tissue. Biological and material design constraints, respectively dictate that MEA devices should not elicit a foreign body response and should be resistant to electrode corrosion and insulation deterioration. Though much current research is focused on biological intervention strategies and hardware advancements to mitigate the biological, material, and mechanical sources of signal instability (for reviews, see Jorfi et al., 2015; Kozai et al., 2015b; Lecomte et al., 2018; Wellman et al., 2018), the time required for iterative redesign, testing, and regulatory approval to translate these improvements to clinical BMIs is substantial. While the neurotechnology field is advancing, even the best neural implants are subject to a range of potential disruptions that affect MEA signals and limit BMI system performance.

A promising alternative approach to counteract signal deterioration is developing algorithmic methods to monitor and compensate for disruptions. One benefit of this approach is its potentially short timeline for development, deployment, and impact. In contrast to the extensive and time-consuming regulatory approval process required for hardware modifications, software can be rapidly implemented and upgraded, conferring immediate benefits to the user. Another advantage of this approach is its inherent flexibility and customization potential. Software can be made to adapt to chronic changes in signal characteristics and tailored to specific users or disruption processes. Lastly, algorithmic strategies are the only means to restore BMI performance following disruptions that affect implanted components because MEA repairs and modifications are not feasible under most circumstances.

When designing algorithmic strategies to mitigate signal disruptions, the underlying cause of a disruption becomes secondary in importance to its impact on recorded signals. With this shift in perspective, it becomes evident that the categorization of disruptions as biological, material, or mechanical needs augmented to include temporal characteristics of the disruption and a sense of whether and how the signal is recoverable. Within each of these three causal categories, disruptions may have vastly different consequences on signal quality. For example, neuroinflammation, glial scarring, and neurophysiological state changes are all of biologic origin but likely impact distinct attributes and time scales of recorded signals. Here, we propose a set of disruption categories that describe the changes of recorded signals and the amenability of those changes to algorithmic compensation. We classify commonly observed disruptions of MEA recordings into one of four groups according to the following definitions:

### Transient Disruptions

Transient Disruptions interfere with recordings on the time scale of hours or less and may resolve spontaneously. However, recorded signals do not necessarily revert to a previous state following a transient disruption.

### Reparable Disruptions

Reparable Disruptions cause persistent interference in recordings that typically does not spontaneously resolve. Good signal quality can be restored with a targeted intervention that addresses the root cause.

### Irreversible Compensable Disruptions

Irreversible Compensable Disruptions cause persistent or progressive reduction in signal quality. While the underlying cause cannot be remedied, the effects may be compensated for algorithmically.

### Irreversible Non-compensable Disruptions

Irreversible Non-compensable Disruptions cause persistent or progressive reduction in signal quality, cannot be remedied by fixing the root cause, and are not amenable to algorithmic compensation. These disruptions indicate severe failures that may render the interface inoperable.

Assigning disruptions into these categories is useful because each category aligns closely with strategies to detect and correct signal disruptions. For instance, adaptive decoding algorithms are well-suited to compensate for the acute shifts in neural recordings caused by transient disruptions. Likewise, algorithms that monitor longitudinal signal quality can detect reparable disruptions such as faulty connections or external cable damage and may provide clues that a user is fighting a systemic infection that requires antibiotics. Irreversible, compensable disruptions, such as the formation of a glial scar or electrode insulation cracking, may be overcome by optimizing neural decoding features in affected channels. Irreversible, non-compensable disruptions such as meningeal encapsulation and ejection of the MEA from the cortex result in widespread signal loss that cannot be recovered with algorithmic strategies. We note that these categories are not entirely mutually exclusive, and some disruptions may fall in more than one category based on severity. Nonetheless, the broad categorization can be used to organize disruptions by performance impact and potential for remediation.

**Figure 2:**
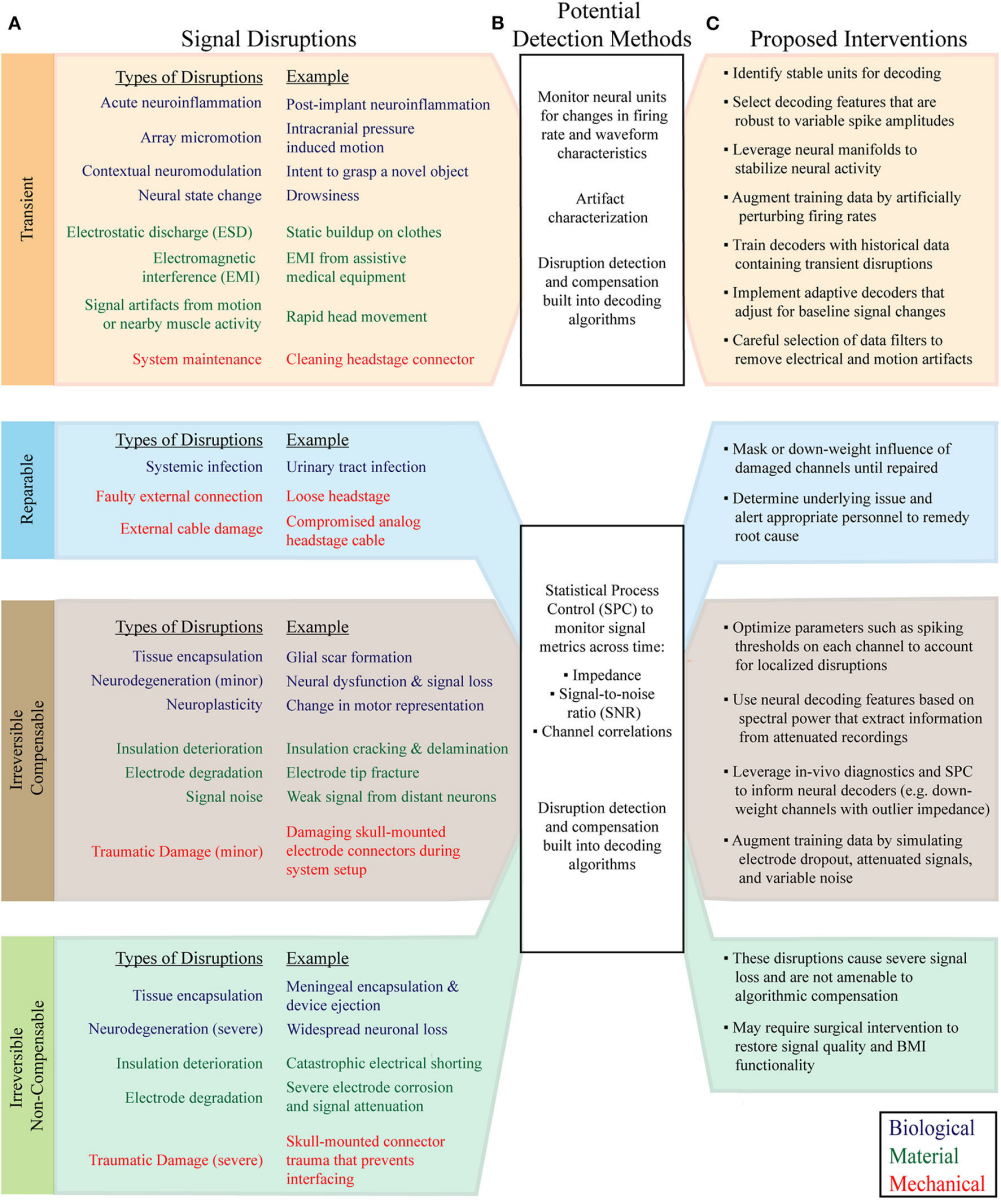
Classification of common MEA signal disruptions and applicable compensatory strategies. **(A)** Signal disruptions are classified according to their underlying cause (Biological, Material, or Mechanical), and impact on signal quality and responsiveness to intervention (Transient, Reparable, Irreversible Compensable, and Irreversible Non-compensable). **(B)** Signal disruptions can be explicitly detected with statistical monitoring of neural features and recording metrics. Following the detection of a disruption, BMIs can initiate tailored algorithmic countermeasures to adapt to changes in signal characteristics. In parallel, advanced machine learning algorithms and decoder training strategies mitigate the effect of disruptions without requiring explicit detection. **(C)** The newly proposed disruption classes have characteristic interventions that help maintain BMI performance. Signal preprocessing, data augmentation, neural feature selection, neural manifolds, and adaptive neural decoders are among the most useful techniques for mitigating the effects of recording disruptions.

In the following sections, we review commonly observed MEA signal disruptions of biological, material, and mechanical etiologies and also demonstrate application of the proposed expanded classification method. We concentrate primarily on disruptions affecting the Utah array because it is currently the gold standard for clinical intracortical BMIs (Hochberg et al., 2012; Klaes et al., 2015; Bouton et al., 2016; Flesher et al., 2016; Ajiboye et al., 2017; Thomas et al., 2020), and both the chronic recording performance and failure modes have been thoroughly investigated. Nevertheless, many of the following disruptions and corresponding algorithmic strategies are applicable to neural interfaces that use other intracortical MEAs. An overview of common signal disruptions with their expanded classifications, potential detection methods, and compensatory strategies is shown in Figure 2. We conclude this review with a discussion of the mitigation strategies appropriate to each of the newly introduced categories.

## Biological Disruptions

Adverse biological reactions to neural implants are well-characterized (for recent reviews see Kozai et al., 2015b; Campbell and Wu, 2018). To date, there has been no functional intracortical multi-electrode recording device that completely avoids the biological responses to implantation that preclude long-term neural recordings. In addition to detailing the effects of chronic biological reactions on signal acquisition, this section reviews the biological sources of acute recording disruptions that can also be detrimental to decoding accuracy.

### Blood Brain Barrier (BBB) Damage

**Figure 3:**
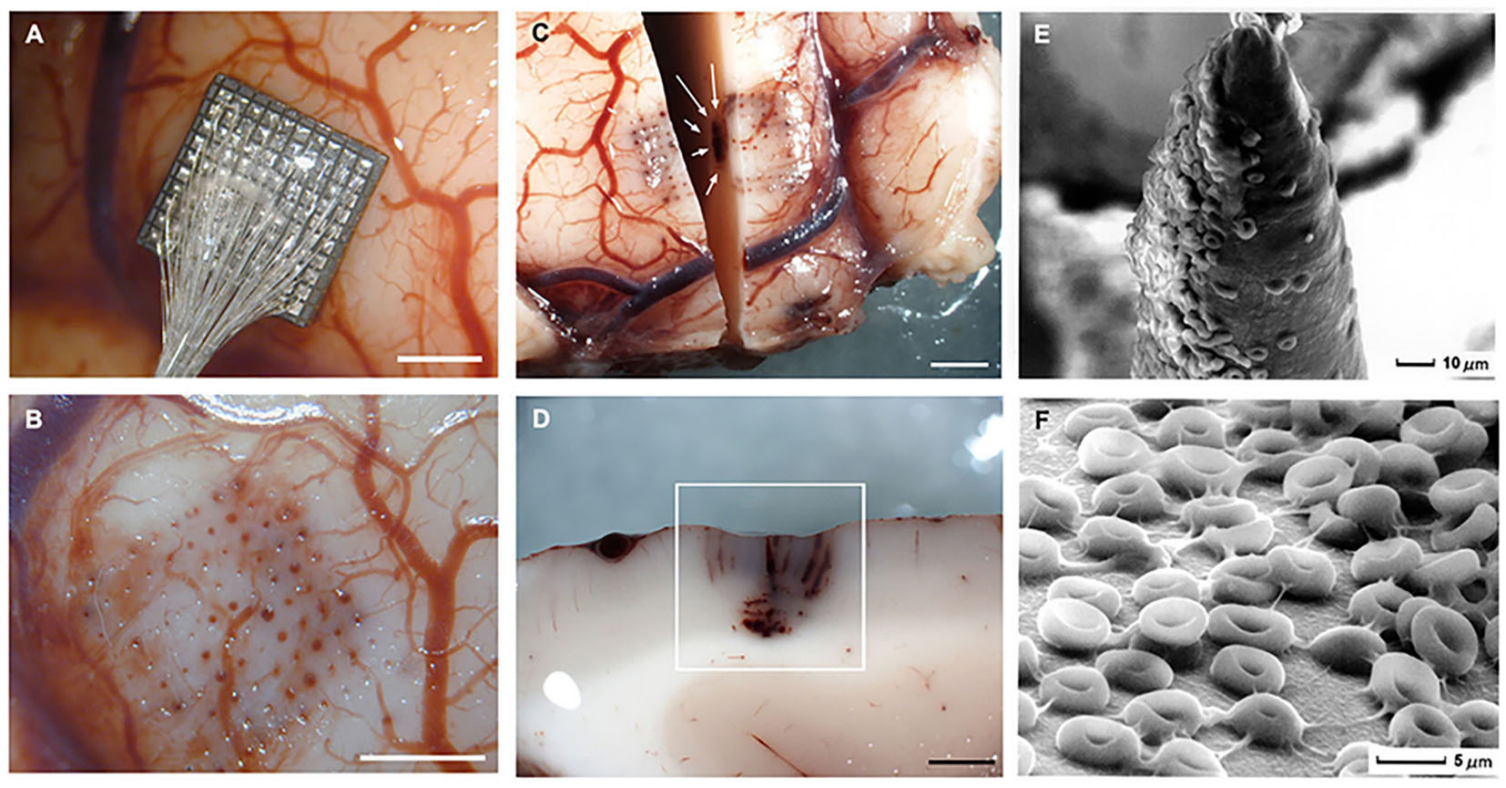
Gross specimens of human temporal lobe implantations and micrographs of the surface of the Utah electrode array after acute implantation in human brain. **(A)** Placement of an electrode array in temporal cortex. **(B)** Once the array has been removed there are some evident microhemorrhages. **(C)** Horizontal section showing blood in the outermost electrode tracks and petechial hemorrhages (white arrows) located below the tip of the electrodes. **(D)** Detail of the petechial hemorrhages. **(E)** Scanning electron micrograph of an electrode tip. Many red blood cells appear in close contact with electrode materials. **(F)** Detail of the red blood cells on the surface of the microelectrodes. Calibration bars **(A–D)** = 2 mm. Figure and caption reprinted from Fernández et al. (2014). Microhemorrhages such these are associated with acute neuroinflammation and transient disruption of signal quality.

Electrode implantation causes trauma to cortical tissues and directly damages the blood-brain barrier. Penetrating electrodes displace local tissue and cause minor cortical tearing in addition to rupturing, severing, and dragging of the microvasculature (Bjornsson et al., 2006; House et al., 2006). Even though MEAs are carefully placed to avoid major vessel trauma during implantation, they inevitably cause microvascular damage because individualized electrode placement around microvessels is not possible. Implantation in the human cortex has caused microhemorrhages around electrode tracks and petechial hemorrhages below electrode tips (Figure 3; Fernández et al., 2014). BBB disruption, evidenced by local increases of ferritin, immunoglobulin, and albumin at the electrode-tissue interface, persists throughout the entire implant duration and is associated with poor recording performance (Prasad et al., 2012, 2014; Saxena et al., 2013; Nolta et al., 2015).

BBB disruption degrades recording quality through several mechanisms. First, the damaged vasculature enables infiltration of proinflammatory macrophages and myeloid cells at the implant site (Saxena et al., 2013). These cells produce cytokines that promote neuroinflammation, enhance BBB permeability, and create a feedback loop that propagates chronic inflammation, neurodegeneration, and signal deterioration (Saxena et al., 2013). Secondly, loss of the BBB facilitates plasma protein leakage into the peri-electrode space, contributing to astroglia and microglia activation, further amplifying neuroinflammation (Kozai et al., 2015b). Erythrocyte infiltration and degradation following microhemorrhages at the implant interface increases free iron levels, which in turn promotes local oxidative stress (Bennett et al., 2019). Lastly, damaged vasculature allows for an unregulated influx of molecules around the array that can disrupt local ionic gradients and synaptic stability, ultimately resulting in variable neuronal responses.

#### Signal Disruptions Due to BBB Damage

##### Transient disruptions

Acute neuroinflammation and homeostatic imbalances cause acute firing rate modulations of neurons recorded by the array as well as changes in background biological noise. These biological responses decrease recording consistency, which can negatively impact BMI decoder performance. Resolution of acute neuroinflammation can reverse these signal changes.

##### Irreversible compensable disruption

Chronic inflammation is associated with minor loss of neurons around the array, resulting in a decrease of available information in the MEA recording. Neurodegenerative states such as these are associated with chronic, slowly progressive increases in neural response variability, dropout of previously recorded units, and decline in signal to noise ratio of recorded signals.

### Tissue Encapsulation

**Figure 4:**
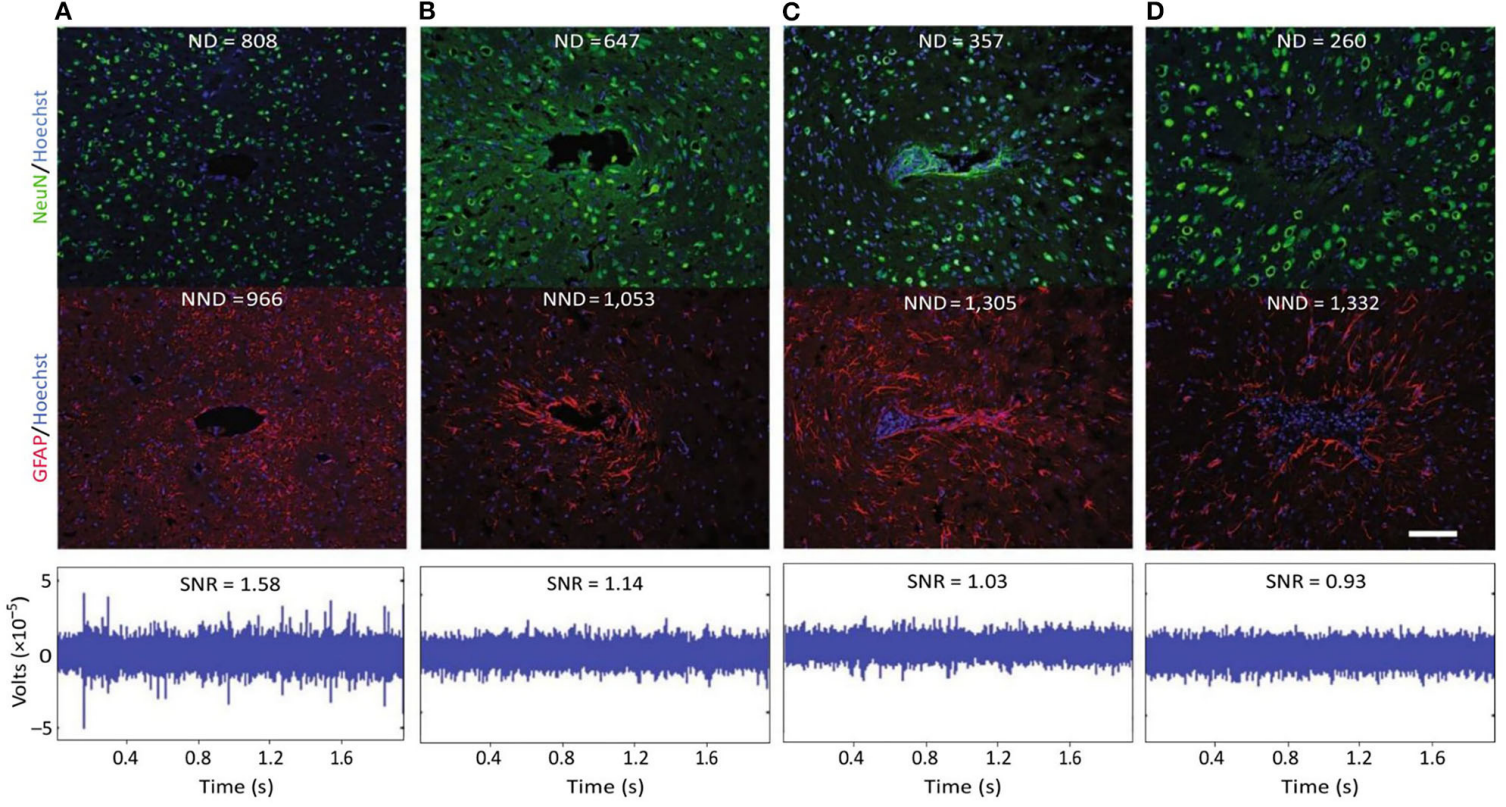
Evidence for a negative impact of increased gliosis on recording quality. **(A–D)** Representative images from four animals demonstrate the range of endpoint histological outcomes (from “good” to “poor,” left to right). Neuronal nuclei (NeuN, green) and astrocytes (GFAP, red) surrounding probe tracts are shown, and the associated average neuronal and non- neuronal density data are listed [area binned cell counts, neuronal density (ND), and non-neuronal density (NND), in cells mm^−2^]. Recording segments with signal-to-noise-ratio (SNR) values representative of the average value for each animal are depicted. Recording quality improved with decreased NND and increased ND/NND (*P* < 0.05, Spearman's ρ, *n* = 6). Impedance increased with increased NND (*P* < 0.05, Spearman's ρ, *n* = 6). Scale bar, 100 μm. This figure was generated after additional analysis on data collected in Purcell et al. (2009). Figure and caption adapted with permission from Salatino et al. (2017). Copyright 2017, Springer Nature. Chronic changes from glial scarring are associated with irreversible, compensable signal disruptions.

Following device implantation, microglia and astrocytes are activated and recruited to the electrode interface where they form a sheath around electrodes (Szarowski et al., 2003; Biran et al., 2007; Kozai et al., 2012, 2015b; Fernández et al., 2014; Salatino et al., 2017). The extent of glial scarring is variable, and selective electrode encapsulation has been observed for neighboring recording sites in the same array (Rousche and Normann, 1998). Such inconsistencies could be due to variations in local tissue and microvascular damage during implantation. Electrodes surrounded by increased densities of non-neuronal cells, including microglia and astrocytes, tend to acquire lower quality signals (Figure 4; Salatino et al., 2017). Furthermore, MEAs are susceptible to fibrous encapsulation that can cause gross array movement, chronic recording instability, and widespread signal loss (Barrese et al., 2013; Woolley et al., 2013; Cody et al., 2018; Eles et al., 2019). Here we discuss several of the mechanisms by which glial scarring and fibrous encapsulation affect recorded signals.

Glial scarring is most likely to disrupt recordings during acute, post-implant scar formation, and tissue stabilization around the implant. This process is commonly identified as the cause for the substantial increase in electrode impedance typically seen within the first weeks after implantation (Williams et al., 2007; McConnell et al., 2009a; Mercanzini et al., 2009; Barrese et al., 2016). Heightened impedance with scar formation suggests that the scar electrically insulates the implanted device and restricts current flow. Converging evidence supports the insulating role of the glial scar, demonstrating that the glial sheath inhibits molecular diffusion (Roitbak and Syková, 1999). In addition, scar formation may influence synaptic transmission and modulate surrounding cellular and neuronal population activity (Salatino et al., 2017), altering MEA signal characteristics as the scar forms. Changes in glial scar morphology have been observed up to 16 weeks post-implant (McConnell et al., 2009b; Potter et al., 2012), highlighting the potential for dynamic changes in the MEA recording environment over this timeframe. Nevertheless, several groups have observed that scar stabilization and chronic decreases in recording quality are not temporally aligned, necessitating the involvement of other failure mechanisms in loss of signal quality (Winslow et al., 2010; Barrese et al., 2013, 2016; Malaga et al., 2016; Black et al., 2018).

Activated glial cells may contribute to chronic signal disruptions by producing proinflammatory cytokines that can lead to neurodegeneration (Salatino et al., 2017). Indeed, high levels of activated glial cells are associated with neuronal loss adjacent to electrodes, which is likely a result of neurotoxic inflammation (Biran et al., 2005, 2007; McConnell et al., 2009b). Furthermore, these scars are known to create a local inhibitory environment that impedes axon regeneration (Fawcett and Asher, 1999). Irreversible neuronal loss decreases the signal-to-noise ratio (SNR) of recorded signals and causes dropout of previously recorded units.

**Figure 5:**
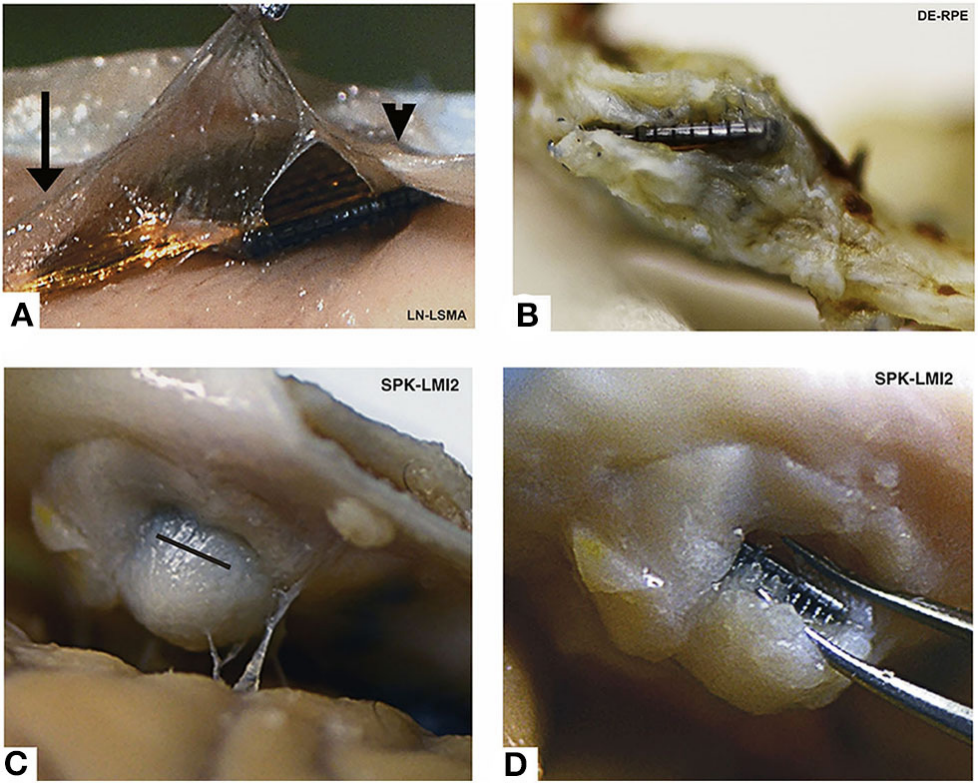
Encapsulated arrays—gross specimens. All arrays show grossly visible encapsulation, however the extent of encapsulation varies greatly. **(A)** Thin tissue capsule with arachnoid appearance at 37 days post-implant. This tissue can be seen merging with normal arachnoid to the left (arrow) and normal dura to the right (arrowhead). **(B)** Dense fibrous tissue encapsulation at 761 days post-implant. The array is intradural in this photo. **(C)** Complete encapsulation by day 853. The capsule was cut open (black line) in order to visualize the array seen in **(D)**. Array names reflect monkey name and implant location. Figure and caption adapted from Barrese et al. (2013), © IOP Publishing. Reproduced with permission. All rights reserved. Chronic changes from meningeal encapsulation can be associated with irreversible, compensable signal disruption when the encapsulation is minor; or irreversible, non-compensable signal disruption when the process is severe.

While the time course and effects of parenchymal encapsulation on recording quality are still being debated, it is generally agreed that meningeal encapsulation is a significant failure mode of intracortical electrodes. In fact, meningeal encapsulation and extrusion of intracortical arrays is the most common chronic failure mode of NHP MEAs (Barrese et al., 2013). Encapsulation occurs when meningeal cells migrate down the implant from the cortical surface and form a capsule that conforms to the implant and thickens over time (Woolley et al., 2013; Eles et al., 2019). Examples of extensive fibrous encapsulation around chronically implanted Utah arrays are shown in Figure 5 (adapted from Barrese et al., 2013). The tissue capsule exerts mechanical forces that can ultimately eject the device from the cortex (Rousche and Normann, 1998; Barrese et al., 2013; Cody et al., 2018). Excessive local meningeal proliferation can also result in a downward pressure that causes indentation of the cortical surface (Rousche and Normann, 1998). In either case, movement of the array changes the depth of the recording sites in the cortex and chronically disrupts signal stability.

#### Signal Disruptions Due to Tissue Encapsulation

##### Irreversible compensable disruptions

Scar formation and stabilization can be associated with increased impedance, reduction in signal amplitudes, and decreased SNR due to electrode encapsulation and neuronal loss. Fluctuations in scar morphology and local neuronal density near the implant cause variability in recorded potentials across time. Minor meningeal encapsulation and gradual array movement may alter spike amplitudes, noise levels, and lead to loss of isolated units. These irreversible changes may nevertheless be compensable via algorithmic strategies.

##### Irreversible non-compensable disruption

Severe meningeal encapsulation and array movement can progress to ejection of the device from the cortex, resulting in complete or near-complete signal loss which may disable the BMI.

### Neuronal Degeneration

Device implantation results in a decrease in local neuronal density, particularly within 50 μm of the electrodes (Biran et al., 2005; McConnell et al., 2009b; Winslow et al., 2010; Potter et al., 2013; Ravikumar et al., 2014; Gaire et al., 2018). As has been previously discussed, neuronal loss is attributable to a combination of traumatic damage during MEA insertion, the formation of a glial scar, and the neurotoxic and pro-inflammatory environment in tissue surrounding the MEA. During the acute post-implant phase (<4 weeks), local neuronal density may be dynamic (Potter et al., 2012). However, at chronic time points (≥12 weeks), evidence for both progressive neuronal loss (McConnell et al., 2009b; Potter et al., 2012), and stable neuronal density (Winslow et al., 2010) have been reported. At least some of this variability appears to be related to the specific method of device sterilization used pre-implantation; one study showed a temporal decline in neuronal density between 2 and 16 weeks only for certain device sterilization techniques (Ravikumar et al., 2014). Because microelectrodes are believed to be sensitive to neurons within 140 μm of the recording site (Buzsáki, 2004), local changes in neuronal viability are likely to substantially affect recordings.

**Figure 6:**
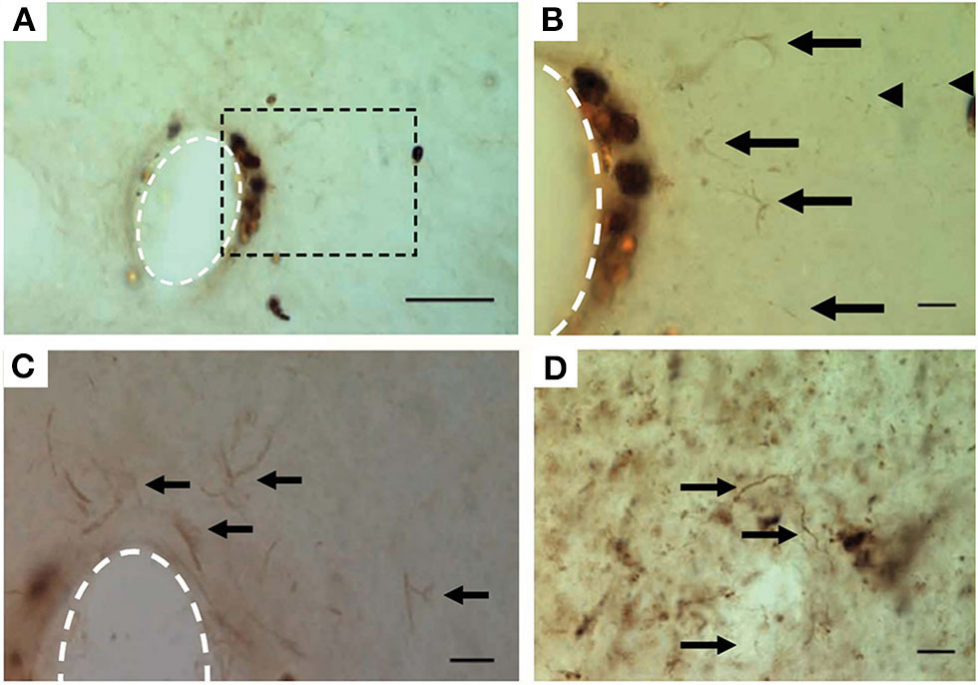
Hyperphosphorylated tau surrounding chronically implanted microelectrodes 16 weeks post-implant. **(A–C)** Representative images of pT231 immunostained pretangles. **(B)** Boxed region in **(A)** at higher magnification. Note the increased presence of pT231 staining near non-specifically stained hemosiderin-laden macrophages. **(C)** Additional example from another implant showing the pT231 positive signal surrounding the electrode. Arrows indicate labeled diffuse granular pretangles and arrow heads indicate rodlike dystrophic neurites. **(D)** Positive control—human Alzheimer's case sections contained stained processes similar to those seen around electrodes. The scale bar is 50 μm **(A)** and 10 μm **(B–D)**. Figure and caption reprinted with permission from McConnell et al. (2009b). © IOP Publishing. All rights reserved. Tau protein misfolding and neuronal degeneration cause irreversible signal disruption that may be compensable with algorithmic strategies.

Neurodegenerative or pathological states have been observed near the implant site as early as 2–16 weeks post-implant (McConnell et al., 2009b; Potter et al., 2013; Saxena et al., 2013). Tau protein pathology, a characteristic form of neurodegeneration that is a consequence of neuroinflammation and microglia activation (Ising et al., 2019), has been found in axons surrounding implanted microelectrodes (Figure 6; McConnell et al., 2009b). Hyperphosphorylated tau causes this intracellular protein to misfold and clump into tangles inside neurons. Multiple lines of evidence suggest that tau protein pathology is associated with alterations in synaptic connectivity, abnormal spontaneous spiking activity, and changes in neuronal firing rates (Frere and Slutsky, 2018), which can contribute to destabilization of neural signals near the implant.

Other factors contributing to neuronal degeneration and dysfunction have also been reported. MEA implantation has been associated with loss of myelin near the electrode interface (Winslow et al., 2010), a condition that impairs signal transduction of affected neurons. Furthermore, local dendritic loss has been reported (McConnell et al., 2009b), which can affect synaptic processing and neuronal excitability (Šišková et al., 2014). Observations of poor recording performance in the absence of both device material failure and severe neuronal loss suggest that some local neurons become impaired or silenced (Michelson et al., 2018). Collectively, these degenerative and pathological states lead to neuronal signaling instabilities that affect both acute and chronic decoding performance.

#### Signal Disruptions Due to Neuronal Degeneration

##### Irreversible compensable disruptions

Chronic neurodegeneration and neuronal dysfunction lead to inconsistent neuronal signaling and the potential for a gradual decline in the number of recorded single units. Although these conditions are irreversible, meaningful signal may still be recoverable through neural decoder feature optimization and other algorithmic strategies.

##### Irreversible non-compensable disruption

In extreme cases of neurodegeneration or tauopathy, there is severe, irreversible signal loss not compensable through algorithmic strategies.

### Inflammation and Infection

Apart from acute neuroinflammation associated with BBB breach, several other factors may cause or exacerbate the local neuroinflammatory response and recording signal disruption. For example, increased levels of residual endotoxins on neural implants after sterilization have been observed to cause greater microglial and macrophage activation, glial scarring, and neuronal loss at the implant site acutely after surgery (Ravikumar et al., 2014). Activated glial cells near the electrode interface produce pro-inflammatory cytokines such as tumor necrosis factor alpha (TNF-α) and interleukin-1β (IL-1β) that can affect neuronal excitability and contribute to a neurotoxic environment (Biran et al., 2005; Karumbaiah et al., 2013; Vezzani and Viviani, 2015). Neural tissue with increased expression of genes encoding for pro-inflammatory cytokines have been linked to reduced SNR in neural recordings (Saxena et al., 2013). In addition, histological and gene expression analyses have demonstrated heightened oxidative stress at the tissue-electrode interface (Potter et al., 2013; Ereifej et al., 2018; Bennett et al., 2019). Reactive oxygen species (ROS), formed by inflammatory cells or during electrochemical reactions at the electrode surface, are known to deteriorate electrode materials, cause neuronal loss or degeneration, and potentiate neuroinflammation (Potter et al., 2013; Potter-Baker and Capadona, 2015; Takmakov et al., 2015), resulting in MEA signal instabilities.

Mounting evidence suggests that stiff mechanical probes propagate local neuroinflammatory cascades. Not only do mechanically stiff probes result in greater micromotion induced stresses (Subbaroyan et al., 2005; Sridharan et al., 2015), but they also decrease BBB integrity, increase glial scar density, increase neuronal loss, and increase levels of activated microglia and macrophages (Nguyen et al., 2014; Du et al., 2017). Thus, stiff materials such as silicon and tungsten, often used to fabricate neural implants, likely exacerbate local neuroinflammation and contribute to signal deterioration.

Lastly, current clinical BMI systems utilize transcutaneous connectors that have local skin sites that are prone to infection. Superficial infections may be treated with topical or oral antibiotics and may not affect MEA signal. However, deep infections spreading to bone that supports the connector could result in loosening of the screws leading to mechanical failure (Fang et al., 2017). In more severe cases, deep tissue infection could require surgical intervention or lead to death (Barrese et al., 2013). In parallel, BMI users with a spinal cord injury or similar disability are at higher risk for systemic infections unrelated to the implant, e.g., urinary tract infections (Garcia-Arguello et al., 2017). Several links between peripheral inflammation and CNS modulation have been identified (Dantzer et al., 2008; Teeling and Perry, 2009), and systemic infection has been anecdotally associated in time with decline in BMI decoder accuracy in clinical trials (Schwemmer et al., 2018). However, formal study of the relationship between BMI performance and systemic infections remain to be undertaken. Evidence that inflammatory responses are exaggerated in those with neurodegenerative disorders (Teeling and Perry, 2009) could mean that signal disruptions due to infection are more pronounced in certain clinical BMI populations than in NHP studies with otherwise healthy subjects.

#### Signal Disruptions Due to Inflammation and Infection

##### Transient disruptions

Acute neuroinflammation or tissue edema after implantation may cause transient changes in firing rate that may resolve spontaneously when the underlying biological processes resolve.

##### Reparable disruptions

Systemic infection is likely to cause altered neural signaling and recording instability that is reversible with systemic antibiotics.

##### Irreversible compensable disruptions

Chronic inflammation is associated with altered neuronal signaling, loss of recorded units, and a decrease in SNR that may be irreversible, but also potentially compensable with algorithmic strategies.

##### Irreversible non-compensable disruption

Severe local deep tissue infections at the MEA implantation site may cause irreversible tissue changes, disruption of neural recording, and may require surgical intervention for device explantation.

### Array Micromotion

Inconsistent neuronal firing rates and spike waveforms from the same MEA channel and subject have been reported in both clinical and NHP trials. One NHP study evaluating motor cortex recordings revealed that 61% of neurons were unstable over 15 days (Dickey et al., 2009). A study in humans reported that 60% of units were unstable after a single day (Downey et al., 2018a). A similar clinical trial showed that firing rates and spike amplitudes varied for 84% and 74% of units, respectively, within a single recording session (Perge et al., 2013).

These instabilities likely arise from two sources: neurophysiological changes (discussed in the following section) and small fluctuations in spatial proximity between electrodes and neurons. One observation that has been interpreted as evidence of micromotion causing signal variability is the synchronous shift in spike amplitudes across the array (Perge et al., 2013). In NHPs, high acceleration head movements have been proposed as a contributing factor to array micromotion, as they have been linked to abrupt changes in neuronal peak-to-peak voltages (Santhanam et al., 2007). However, different micromotion mechanisms may be at work in human studies, as severe signal instabilities have been identified in humans in the absence of rapid head movements (Perge et al., 2013). Additionally, abrupt electrode shifts are not consistent with the gradual loss of stable units observed in humans (Downey et al., 2018a). Alternative explanations for micromotion that may be more common in clinical trials include changes related to intracranial pressure, local vasculature, or biological processes occurring at the tissue-electrode interface.

**Figure 7:**
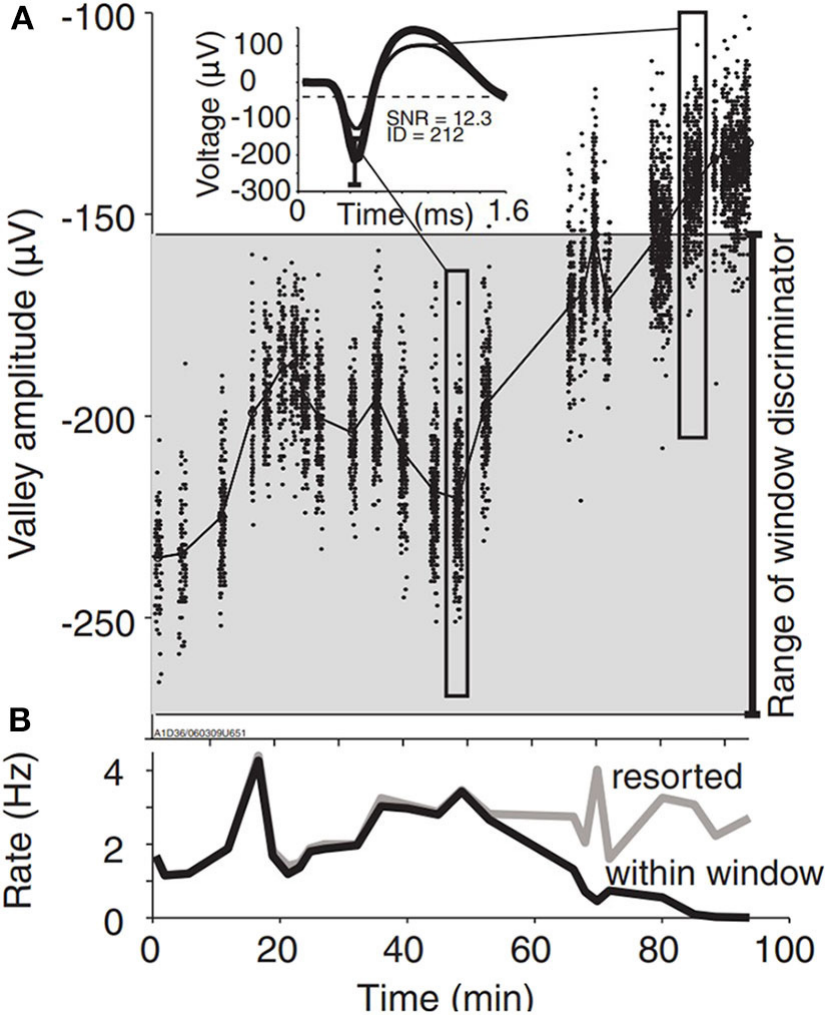
Spike amplitude instability causes spike detection error. **(A)** A representative unit demonstrates large spike amplitude instability. The gray shaded area covers the amplitude range between the upper and lower boundaries of the window discriminator as determined manually by the experimenter. Spikes falling outside of these boundaries remained undetected during the online experiment. Inset: average spike waveforms during selected time periods indicated by elongated rectangles. **(B)** Spike rates as determined by online (within window, black) and retrospectively discriminated spikes (resorted, gray). Apparent decline in the online firing rate results from failure of the smaller waveforms to satisfy the discriminator parameters. Figure and caption reprinted with permission from Perge et al. (2013). © IOP Publishing. All rights reserved. Courtesy of braingate.org.

Small shifts in array location may cause small changes in waveform amplitude (i.e., spike amplitude instability) that translate into significant impacts on apparent spike rate and BMI decoding performance. Perge et al. provide an illustrative example of spike detection error caused by a rapid baseline shift to 44% smaller spike amplitudes. This was interpreted as a 50% drop in the unit's apparent firing rate because the spikes no longer met predefined amplitude criteria for the thresholding process (Figure 7). Interestingly, offline spike resorting revealed that the unit actually increased firing rate during this time (Perge et al., 2013).

#### Signal Disruptions Due to Array Micromotion

##### Transient disruption

Array micromotion may cause apparent changes in neuronal firing rates and spike amplitudes on the time scale of minutes to hours. Adaptive thresholding algorithms may help identify these situations and mitigate their effect on BMI performance.

### Neurophysiological Changes

Acute changes in recordings may also result from neurophysiological changes in the recorded neuronal population. Perge et al. reported that ~85% of the observed instability in units was due to variability in spike generation, citing cognitive and behavioral changes, neural plasticity, or other unknown physiological mechanisms as likely factors (Perge et al., 2013). Intracortical recording variabilities in somatosensory or motor areas have been associated with the subject's emotional state (Kennedy, 2011), attentional state (Steinmetz et al., 2000), and arm posture (Scott and Kalaska, 1995). Neuroplastic changes from BMI practice or other neurorehabilitation methods may also alter neural representations across longer time scales (Sanes and Donoghue, 2000; Dobkin, 2007; Ganguly and Carmena, 2009). It is known that EEG recording is sensitive to acute variations in neural activity associated with medications, psychoactive substances such as caffeine, and physical and mental fatigue effects. It is not clear, however, to what extent these variations are represented at the level of MEA recording, e.g., in the hand/arm area of the motor cortex.

Other changes in firing rate may contain important information about context. For example, researchers investigating human motor cortex activity during control of a grasp neuroprosthetic reported that firing rates shifted in the presence of an object to be grasped (Wodlinger et al., 2015; Downey et al., 2017). Therefore, in order to successfully perform BMI-controlled object interaction tasks, BMI decoding algorithms must account for the changes in neural firing attributed solely to the presence of the object as well as neurophysiologic changes associated with the user's intent to grasp it (Wodlinger et al., 2015; Downey et al., 2017).

Researchers have also shown that during reaching tasks, neural activity encodes not just arm kinematics, but also distinguishes between being in a state of rest vs. holding a static reaching position (Velliste et al., 2014). These changes in neural population tuning are important context-based signal disruptions that can interfere with prosthetic use and generate non-zero velocity predictions during rest if not recognized and properly handled (Velliste et al., 2014). Currently, most BMIs are operated in a controlled laboratory setting, thus minimizing contextual variability from session to session. However, if used as assistive devices in everyday life, BMIs will be used in broader and potentially unpredictable circumstances, substantially contributing to context variability in neural representations.

Regardless of the underlying mechanisms, acute recording instabilities have the potential to negatively impact BMI decoding performance. In fact, firing rate instabilities have been shown to create a directional bias during cursor control strong enough to decrease target acquisition from 100% to chance levels in as little as 30 min (Perge et al., 2013). To prevent such dramatic decreases, some groups have proposed utilizing adaptive decoders that can update their parameters to account for instabilities. However, additional instability may result as the user continuously adapts to a regularly updating decoding model. An optimal balance between decoder adaptation and neural adaptation may improve BMI performance and robustness to disruptions (Shenoy and Carmena, 2014). Ultimately, the deployment of portable intracortical BMIs will be critical in determining the extent to which contextual and other physiological factors impact functional BMI performance.

#### Signal Disruptions Due to Neurophysiological Changes

##### Transient disruption

Changes in emotional, cognitive, environmental or physical states may cause acute variation in neuronal firing rates on the time scale of minutes to hours. Using adaptive machine learning decoders trained on substantial historical data may make BMIs robust to context-specific neural features.

##### Irreversible compensable disruption

Neuroplasticity associated with learning and practice may induce chronic, irreversible changes in neural representations that are compensable with algorithmic strategies.

## Material Disruptions

Intracortical arrays are subject to ongoing biologic reactions that continually deteriorate device components. Explanted arrays exhibit evidence of these morphologic changes, which generally increase in severity with indwelling time. MEAs are susceptible to a variety of sources of transient and persistent noise whose effects can be exacerbated by material failures, e.g., damaged insulation or connector devices. These material disruptions act synergistically to degrade signal quality.

### Pre-implant Failure

Microelectrode array fabrication is an imperfect process, and defects have been noted even before the devices are exposed to the harsh *in vivo* environment. Material defects not only increase the risk of signal attenuation and corruption, but also prime the array for other sources of failure. For instance, the manufacturing inconsistency of planar silicon electrodes is thought to partly explain variability in mechanical failure (Kozai et al., 2015a). Pre-implantation scanning electron microscopy (SEM) of Microprobes parylene-C coated platinum/iridium (Pt/Ir) arrays reveal non-uniform insulation with substantial cracking, as well as bent or cracked recording sites, which together affect ~25% of the total electrodes (Prasad et al., 2014; Takmakov et al., 2015). In contrast, pre-implant SEM of Blackrock Microsystems Utah arrays indicate only minor insulation delamination and irregularities (Takmakov et al., 2015; Barrese et al., 2016). However, defective internal components may not be apparent with imaging techniques. Instead, these pre-implant failures may be detected by outliers in impedance spectra (Takmakov et al., 2015).

Though great care is taken to reduce the likelihood of manufacturing defects and physical damage during implantation, clinical-grade MEAs are still at risk of these irreversible disruptions. For instance, immediately following MEA implantation for a clinical BMI system, a small fraction of electrodes was identified as non-functional (Simeral et al., 2011). The precise etiology of this failure remains unknown, but it is possible electrodes were damaged while handling the array just before implantation (House et al., 2006) or by forces incurred during cortical insertion. In other, more severe cases, manufacturing defects, and improper sterilization techniques have caused complete array failure in NHPs (Barrese et al., 2013). In conclusion, pre-implant disruptions are rare in clinical-grade devices; however, because they interfere with recordings indefinitely and can degrade signals by accelerating other failure mechanisms, they are still of high importance.

#### Signal Disruptions Due to Pre-implant Failure

##### Irreversible compensable disruption

A limited number of damaged or dysfunctional electrodes may irreversibly distort signals or cause loss of signal from individual channels. These disruptions may be compensable with algorithmic strategies to exclude or down-weight bad channels.

##### Irreversible non-compensable disruption

Severe material defects during manufacturing have potential to cause irreversible, widespread signal loss that is not compensable algorithmically.

### Insulation Deterioration

**Figure 8:**
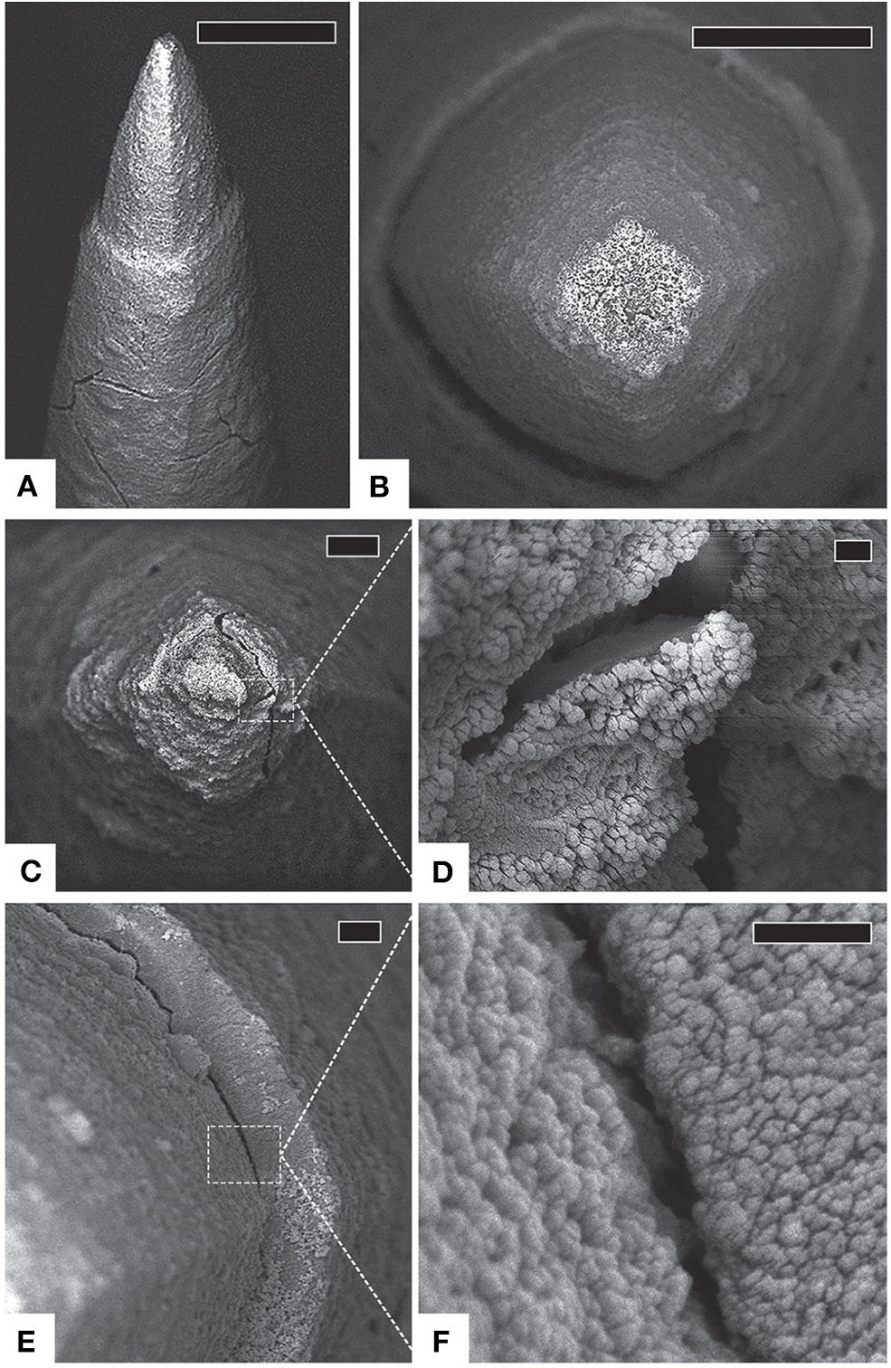
Insulation deterioration and tip cracking. **(A)** Side view of a typical electrode with intact platinum [tip], cracked parylene [shaft], and substantial fibrosis, scale 20 μm. **(B)** Top view of another typical electrode with intact platinum and thick, uniform fibrosis encapsulation, scale 10 μm. **(C)** An electrode tip with thick encapsulation tissue, scale 3 μm. **(D)** Detail of cracked platinum tip, scale 200 μm. **(E)** Delaminating parylene interface, scale 1 μm. **(F)** Detail of parylene delamination, scale 200 nm. Figure and caption reproduced with permission from Barrese et al. (2016). © IOP Publishing. All rights reserved. Tip cracking and insulation deterioration are irreversible, but potentially compensable with algorithmic approaches.

Over a dozen failure modes of microelectrode insulation have been identified (Schmitt et al., 1999). Insulation deterioration creates defects that increase effective conductor surface area. Because the recorded voltage is the average potential across the exposed area, defects can attenuate signals by averaging in weak neural activity from distant sources (Wellman et al., 2018). Moreover, defects allow signal contamination by off-target cells and create low-impedance shunting paths to the local environment, further reducing voltage amplitudes (Caldwell et al., 2018). SEM of explanted Utah arrays after chronic implantation show insulation deterioration, irregular parylene-C and platinum (Pt) interfaces, delamination, and cracking along the electrode shaft (with minor tissue invasion), and delamination near the silicon-coated wire bundle (Figure 8; Gilgunn et al., 2013; Barrese et al., 2016). Both cracking and delamination at the base of Utah arrays have been observed after explant (Kane et al., 2013) and accelerated aging with ROS (Takmakov et al., 2015). Similar or worse degradation is common in alternative devices, regardless of the insulating material (Prasad et al., 2012, 2014; Takmakov et al., 2015). However, more severe signal disruption effects of dielectric damage are expected for neural implants that utilize the same material for both the electrode and conductor because defect sites are equally capable of signal transmission (Caldwell et al., 2018).

MEA insulation is also susceptible to water absorption and infiltration at the electrode boundary. Water absorption negatively affects dielectric properties and leads to signal attenuation and electrical coupling to adjacent traces (Seymour et al., 2009; Wellman et al., 2018). Absorption decreases impedance and increases phase at low frequencies (Xie et al., 2011; Takmakov et al., 2015). Furthermore, water penetration can swell insulation, generate intrinsic stresses, and facilitate conductor corrosion, ultimately reducing dielectric adhesion, and promoting delamination (Schmitt et al., 1999; Seymour et al., 2009; Xie et al., 2011; Gwon et al., 2016). For these reasons, dielectrics with low rates of water absorption are preferred insulators in neural interface applications (Wellman et al., 2018). Apart from tissue-electrode interface disruptions, instances of complete array failure have been attributed to infiltration of water or other fluids at sites including external connectors (Barrese et al., 2013).

*In vivo* cyclic voltammetry (CV) and impedance spectroscopy can help identify current leakage pathway formation. Both the electrode yield and the number of recorded units are negatively correlated with cathodic charge storage capacity, suggesting that device integrity directly affects recording performance (Black et al., 2018). Linear increases in the cathodic charge storage capacity over time suggest that deterioration such as cracking or delamination are ongoing processes throughout the indwelling period of the device (Kane et al., 2013; Black et al., 2018). Additionally, irregularities in CV plots can help identify failures such as iridium oxide film delamination on stimulating electrodes (Troyk et al., 2004). However, the safety and feasibility of CV as a diagnostic tool for the MEAs currently used in human studies is uncertain. Stimulation parameters must be tightly controlled to prevent electrode degradation, neural tissue damage, and undesired neural activation (Cogan, 2008). Furthermore, disruptions to the reference electrode can result in dangerously high currents (Kane et al., 2013). Aside from safety concerns, measurements can be prohibitively time consuming, especially at slow scan rates for MEAs with many electrodes.

In contrast to CV, impedance measurements are easily and regularly obtained during clinical BMI recording sessions to asses recording and stimulating capabilities (Simeral et al., 2011; Zhang et al., 2018; Hughes et al., 2020). Impedance characterization of devices has historically been reported at 1 kHz because it provides information about the exposed electrode area (Hsu et al., 2009) and roughly matches the frequency of an action potential. Importantly, 1 kHz impedance can correlate with recording metrics including array yield and the number of recorded units (Prasad and Sanchez, 2012; Black et al., 2018), with several studies supporting the notion that considerable information about device integrity exists at higher and lower frequencies (Takmakov et al., 2015; Caldwell et al., 2018; Straka et al., 2018). For the Blackrock Utah array with platinum recording electrodes, 1 kHz impedance <60 kΩ indicates shunting to ground (Barrese et al., 2013). Active declines in impedance may signify ongoing insulation deterioration, formation of shunting pathways, and attenuation of recorded signals. Conversely, impedances of several MΩ indicate broken signal paths due to hardware failures or connection disruptions (Simeral et al., 2011). Ultimately, information extracted from impedance measurements could be used to customize signal preprocessing and inform neural decoders to maintain long-term BMI performance despite signal disruptions caused by chronic material failures.

#### Signal Disruptions Due to Insulation Deterioration

##### Irreversible compensable disruptions

Insulation failure can lead to irreversible signal disruptions including reduced signal amplitudes, off-target cell recording, and increases in crosstalk. Signal loss on select channels due to electrode shorting is also possible.

##### Irreversible non-compensable disruption

Catastrophic materials degradation or electrical shorting can result in irreversible, extensive and non-compensable signal loss. Impedance spectroscopy can help identify material degradation and implant failures.

### Electrode Degradation

Electrode materials in clinical intracortical BMIs are either platinum (Pt; for recording) or iridium oxide (IrOx; for stimulation). SEM imaging of explanted Utah arrays generally show limited platinum degradation for recording devices implanted <2 years (Gilgunn et al., 2013; Barrese et al., 2016). At time scales approaching 1,000 days, platinum corrosion, cracking, and peeling have been observed (Figure 8), although some damage likely results from forces incurred during surgical explant (Barrese et al., 2016). Nevertheless, the platinum-coated electrodes on Utah arrays appear more stable than tungsten electrodes which are known to corrode over shorter periods and produce toxic metal ions in the process (Patrick et al., 2011; Prasad et al., 2012). Also, Utah array electrodes appear to withstand bending and buckling better than Pt/Ir electrodes on similar microelectrode arrays (Prasad et al., 2014).

Electrode failures that increase effective surface area, such as cracking or corrosion, decrease impedance, and attenuate recorded signals by averaging voltages over a larger geometric area (Wellman et al., 2018). Delamination is a concern for MEAs with metallic or conductive polymer electrode films, especially under neural stimulation conditions (Cogan et al., 2004; Cui and Zhou, 2007; Boehler et al., 2017; Caldwell et al., 2018). Damage to these outermost electrode films exposes the underlying conductor which can lead to corrosion and further undermining of the film (Caldwell et al., 2018). Corrosion byproducts may decrease signal quality by promoting local inflammation (see section Inflammation and Infection). In severe cases, delamination causes the electrical properties of the exposed conductor to dominate at the recording site, often dramatically increasing impedance and decreasing signal quality (Cui and Zhou, 2007; Boehler et al., 2017; Caldwell et al., 2018). In summary, optimal electrode materials can prolong high-quality signal acquisition, but over chronic periods, current devices are susceptible to electrode degradation that negatively impacts electrical properties.

#### Signal Disruptions Due to Electrode Degradation

##### Irreversible compensable disruptions

Damaged electrodes may cause irreversible distortion or loss of signal that may be compensable through algorithmic strategies.

##### Irreversible non-compensable disruption

Catastrophically damaged electrodes can result in irreversible, extensive and non-compensable signal loss and array failure.

### Signal Noise

Intracortical recording systems are susceptible to both biotic and abiotic sources of noise. Major sources of biotic noise include ionic activity from “background” neurons firing, nearby muscle activity, and motion artifact. First, microelectrodes are sensitive to neurons within ~140 μm of the recording site (Buzsáki, 2004; Moffitt and McIntyre, 2005). Thus, signals acquired from a single electrode could be influenced by dozens or hundreds of neurons depending on implant location, local neuronal viability, and degree of tissue encapsulation. Activity from distant neurons is difficult to effectively isolate and therefore has traditionally been considered signal noise (Lempka et al., 2011; Lopez et al., 2012). As such, changes in neuronal density and firing rates contribute to non-stationary biological noise (Lempka et al., 2011). Secondly, abrupt motions or nearby muscle activity can produce artifacts in recorded signals that are common across all electrodes. Paralikar et al. provide evidence of common noise with similar characteristics to neural activity and demonstrate that traditional noise rejection methods such as differential referencing can be inadequate (Paralikar et al., 2009). Similar deficiencies in eliminating motion artifact have been noted for common averaging referencing as well (Michelson et al., 2018). Finally, the degree of local tissue resistivity from device encapsulation is expected to correlate with thermal noise (Lempka et al., 2011).

Abiotic noise arises from BMI hardware and environmental interference. Contributions from recording systems include electrode-electrolyte interface noise and electronic thermal and flicker noise (Hassibi et al., 2004; Yang et al., 2009; Lopez et al., 2012). High-density arrays are particularly susceptible to cross-talk, which can attenuate recorded potentials and influence signals in adjacent electrodes (Wellman et al., 2018). Future systems that employ high-density arrays and wireless data transmission will also have to contend with hardware constraints that limit data bandwidth. Consequently, these systems may need to employ strategies such as lossy data compression that degrade signal quality.

Environmental noise primarily presents as electromagnetic interference (especially at 60 Hz), but other artifacts such as electrostatic discharge may occasionally disrupt recording. BMI systems that incorporate functional electrical stimulation (FES) to restore hand or arm function (Bouton et al., 2016), or intracortical microstimulation for somatosensory feedback (Weiss et al., 2019), are particularly susceptible to extreme levels of electrical artifact. Large voltage transients during such stimulation periods decrease neural decoding performance in the absence of compensatory algorithms (Young et al., 2018; Weiss et al., 2019). Even in cases where the sources of noise are small in magnitude compared to recorded action potentials, the cumulative effect ultimately acts to lower SNR and decrease neural decoding performance. As BMI systems grow more complex to support multiple and more diverse end-effectors, and are used in new environments, recorded noise levels will continue to become more variable.

#### Signal Disruptions Due to Signal Noise

##### Transient disruptions

Sources of noise, including electrostatic discharge, stimulation transients, and motion artifact commonly cause transient signal artifacts. Contextual environmental noise may also variably influence recordings. These sources of noise can frequently be cleaned from the signal using algorithmic methods.

##### Irreversible compensable disruption

Background neural activity can introduce irreversible signal noise that cannot be robustly isolated but can be mitigated through careful neural feature selection and algorithmic strategies.

##### Irreversible non-compensable disruption

Recording and effector devices are sources of irreversible, inherent noise that are not amenable to algorithmic compensation.

## Mechanical Disruptions

Neural recording systems are susceptible to mechanical interferences at both micro- and macroscopic levels. At the microscopic level, micromotion of the array and mechanical agitation of surrounding tissue are the dominating disruptive modes. However, the mismatch of mechanical properties between the cortex and implants generally manifest as biological disruptions through neuroinflammation, and as such, are covered in previous sections. At the macroscopic level, hardware failures such as faulty connections or physical trauma could rapidly change recordings or cause permanent dysfunction.

### Traumatic Damage

Intracortical MEAs in clinical recording systems currently require a transcutaneous, bone-anchored port to transmit data. Cables that connect to the port have a tall rigid base that can act as a lever to produce large, destructive forces on the connector and skull. Accidental trauma to the connector or forces applied by the cable could result in unrecoverable damage to the system or user. Acute traumatic damage to intracortical MEA systems is the most common failure mode for NHPs (Barrese et al., 2013). While the connector design has improved over time to reduce the likelihood of breaking, the devices are not impervious to mechanical damage. Concerns about mechanical reliability of skull-mounted connectors have prompted the design of accessory hardware to enhance connector stability (McMorland and Velliste, 2013). MEA connectors are also susceptible to localized physical damage that can affect recording channels. For instance, in recent Blackrock designs, the surface of the skull-mounted CerePort connector has an exposed gold connector pin for each electrode in the array. These pins are prone to irreparable damage caused by contact with headstage guide pins and other objects. Utilizing fully implanted systems would reduce the opportunity for external mechanical damage, but design challenges regarding power consumption, device size, and data transmission bandwidth must be resolved to successfully translate fully implantable technology for human BMI applications (Leber et al., 2019). Furthermore, implanted hardware in BMI systems are also at risk for traumatic damage. Components such as electrode traces may suffer mechanical damage, particularly at high-strain areas caused by mechanical property mismatches or device geometry (Kozai et al., 2015a). Lastly, cases of head trauma may disrupt recording by damaging neurons and microvasculature near an implant. Traumatic brain injuries can initiate neuroinflammation, alter intracranial pressure, contribute to chronic neurodegeneration (Mckee and Daneshvar, 2015), and significantly alter the landscape of recordable neurons. Though the incidence of traumatic damage to BMIs can be mitigated by cautious behavior, operating BMIs in uncontrolled environments will increase the risk of these disruptions.

#### Signal Disruptions Due to Traumatic Damage

##### Irreversible compensable disruption

Irreversible signal distortion may occur due to minor damage of irreplaceable hardware components such as external gold electrode pins. Distortions may be compensable with algorithmic approaches.

##### Irreversible non-compensable disruptions

Traumatic damage to the skull mounted connector or internal wire bundle can cause irreversible, non-compensable disruption, and inability to record signals. Head trauma may also result in irreversible neural dysfunction depending on the severity, and it is unclear how algorithmic techniques could improve signal quality after these events.

### Connection Failures

After neural activity is acquired through the microelectrodes on the array, the signals are transferred through a series of cables and connectors, each of which has potential to fail independently. For example, in current clinical BMIs, the filament interface between the CerePort and headstage can accumulate debris that prevents proper interfacing and corrupts signals. Analog headstages are particularly susceptible to noise and can require complicated amplifier connectors to support high numbers of recording channels. Improvements in connection reliability and signal noise can be achieved with headstage hardware that digitizes neural signals near the recording site (Weiss et al., 2020). These digital headstages are also more compact and less obtrusive—factors that may enhance their integration in portable BMI systems (Weiss et al., 2020). Other emerging technologies utilize active circuits to amplify, filter, multiplex, and digitize neural signals directly onboard the implanted device (Jun et al., 2017; Fiáth et al., 2018; Angotzi et al., 2019). On-chip signal processing and digitization not only reduces the likelihood of connection disruptions by circumventing the need for complicated analog headstage connectors, but it also limits interference from noise and movement artifacts by digitizing signals closer to the source. These devices enable recording from hundreds or even thousands of channels (Putzeys et al., 2019), though they have yet to be validated in clinical studies.

**Figure 9:**
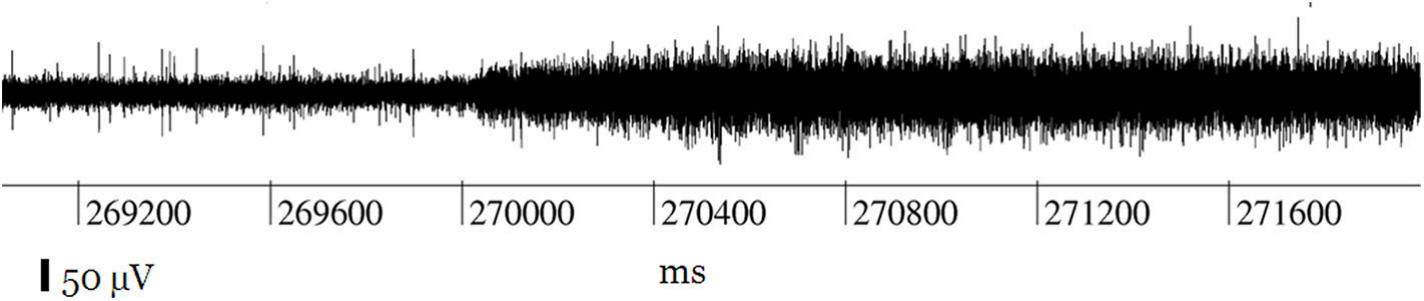
Record of a channel showing the typical effect of connection disruption leading to noise during neural recording: the spikes are lost in increased background noise. Figure adapted with permission from Krüger et al. (2010). Connection disruptions are reparable with engineering intervention.

Literature characterizing how connection disruptions manifest in recorded data is rare. One study, using custom microwires to record from a macaque cortex, reports that impurities between the connector and head stage caused poor contact that resulted in a two-fold increase in noise and the disappearance of spikes (Figure 9; Krüger et al., 2010). The authors note that the contact could open and close dynamically depending on animal movement. After cleaning one of the connectors, they were able to recover ~20 malfunctioning channels. While substantial differences exist between this system and current clinical BMIs, this study highlights how system maintenance can unexpectedly impact recordings. For example, if training data for a decoder were collected using a compromised cable, hardware maintenance may alter recorded signals and ultimately decrease BMI performance. Connection disruptions can also cause high variability in electrode impedance measurements and impair recording consistency (Simeral et al., 2011; Hughes et al., 2020). Although many connection disruptions can be remedied by a technician or a replacement part, further damage is possible during system repair. For instance, improper cleaning of CerePort contact pads has caused recording system failures (Barrese et al., 2013).

Faulty connections are often overlooked as significant failure modes for BMIs, but as these systems become portable and are used without technician oversight, the severity and likelihood of connection disruptions increases. Any system dependent on connection hardware is at risk for faulty connections, cable damage, or hardware malfunction that could interfere with signal transmission. It is important to note that connection disruptions are possible every time the user connects to and disconnects from the system. Given the potential for signal disruptions to be masked during signal processing, e.g., utilizing normalization methods or insensitive feature extraction, it is critical to establish careful data checks as standard operating procedure for device use. Furthermore, especially for clinical BMI systems with stimulating microelectrodes, safety procedures require identification of connection disruptions to appropriately disable electrodes and prevent irreversible damage from exposure to high voltages (Hughes et al., 2020).

#### Signal Disruptions Due to Connection Failures

##### Transient disruptions

Unstable connections may cause temporary loss or gain of viable recording channels. Hardware maintenance may promote the recovery of viable channels.

##### Reparable disruptions

Faulty external cables or connections can cause persistent channel crosstalk, interference, or signal loss. These disruptions can be corrected through repair or exchange of the faulty hardware.

## Discussion

This review has discussed how common MEA signal disruptions of biological, material or mechanical etiologies can further be classified according to their duration and amenability to repair or compensation. This shift in focus from the cause of disruption to characteristic effects on signal and BMI performance provides opportunities to consider how each type of disruption is best identified and what interventions might enable recovery of high-quality signal. Intracortical MEAs are subject to a dynamic *in vivo* environment which, if not accounted for, will render static neural decoders ineffective over relatively short periods. The potential for signal disruptions will further increase as BMI systems transition from being experimental devices used in controlled laboratory settings to portable devices used in the multiple unpredictable environments of daily life. Neural recordings will be subject to unique and varied sources of environmental noise, while hardware components will be at risk of interference, physical damage, and unanticipated challenges in novel use cases. Additionally, the cognitive state of the user and the context in which the device is operated will be highly variable, affecting neural responses in unpredictable ways. Several of these disruptions can be mitigated or even eliminated by improving the materials and design of the neural interface itself, but it is unrealistic to expect that hardware improvements alone can solve this problem. Fortunately, recent developments in machine learning and statistical methods hold promise in mitigating the diverse range of signal disruptions encountered by BMIs. We first consider *in vivo* diagnostics and algorithmic approaches to detect ongoing signal disruptions, followed by a discussion on strategies to combat transient, reparable, and irreversible compensable disruptions.

### Disruption Detection Methods

Identifying ongoing disruptions is an essential step in developing targeted algorithmic countermeasures. One useful diagnostic tool, *in vivo* impedance spectroscopy, has revealed unique impedance signatures for varying degrees of microelectrode tissue encapsulation (Williams et al., 2007; Cody et al., 2018). Impedance spectroscopy, in combination with equivalent circuit modeling, can provide insight on abiotic failure modes such as insulation deterioration, wire breakage, and electrode tip degradation (Caldwell et al., 2018; Straka et al., 2018). Cyclic voltammetry is another method that has proven useful in identifying the formation of current leakage pathways. Indeed, Black et al. found that cathodic charge storage capacity increased with implant time and negatively correlated with electrode yield and the total number of units recorded (Black et al., 2018). However, concerns about the safety and feasibility of this technique with state-of-the-art MEAs in humans has prohibited its translation to clinical studies.

In practice, disruptions can coincide and have overlapping effects that confound diagnostic metrics. For instance, tissue encapsulation of MEA electrodes raises impedance, while insulation deterioration creates shunting paths that lower impedance. Though some disruptions may occur over characteristic time periods (e.g., insulation water absorption and tissue encapsulation following device implantation), compounding effects make it challenging to determine underlying failures precisely. Nevertheless, relationships between impedance and common device failures raise the possibility that researchers could leverage *in vivo* diagnostic techniques to predict recording channels that attenuate signals, or channels that are likely to worsen with time. These predictions could then be utilized when selecting neural features such as channel-wise spike amplitude thresholds. It is also feasible that these predictions could inform decoding models to maintain performance over prolonged periods.

Automated real-time monitoring of signal quality will be a critical component of fielded BMI systems. One potential candidate for signal quality monitoring is statistical process control (SPC) (Western Electric Company, 1956). SPC can be applied in a BMI context by monitoring signal metrics such as impedance, channel correlations, and SNR, and checking for deviations from baseline as well as outlier channels that may indicate hardware failures. For example, insulation degradation can lead to electrical shunting, which may be detected by abnormally high correlation between adjacent channels (Flint et al., 2016). Monitoring impedance is useful for detecting several disruptions, ranging from irreversible electrical shorting due to severe materials degradation, to reparable disruptions such as a loose headstage connector (Simeral et al., 2011; Barrese et al., 2013). Following the automated identification of abnormalities by SPC algorithms, technicians could be alerted, and decoders could be updated to compensate for channels exhibiting abnormal behavior.

BMIs may also leverage statistical approaches to detect transient disruptions such as array micromotion that cause rapid, unexpected changes in firing rates and spike amplitudes. Similar to irreversible and reparable disruptions, early detection of transient disruptions could initiate neural decoder adjustments to mitigate the effects on BMI performance. Furthermore, dramatic drops in BMI performance in the absence of statistical outliers may indicate deficiencies in signal processing and decoding. To our knowledge, there are currently no intracortical BMI systems that implement online disruption detection. Further developing these methods is an avenue for future research. Ultimately, these signal monitoring approaches will help ensure BMIs are functioning properly for extended periods of time and will quickly identify problems that may require intervention.

### Algorithmic Strategies for Transient Disruptions

BMI operation may be influenced by recording instabilities including array micromotion and transient noise, as well as physiological factors such as cognitive or contextual changes that affect intrinsic spike generation (sections Array Micromotion and Neurophysiological Changes). Even in well-controlled environments, BMI performance may continually degrade because of gradual changes in spike rates and signal amplitudes from unstable units. Recent efforts to improve BMI performance have focused on reducing the effects of these transient disruptions and eliminating the need for regular system recalibration. In the following, we discuss neural feature engineering, neural decoder training strategies, adaptive neural decoding methods, and signal filters and referencing techniques that can assist in mitigating the effects of transient disruptions. We discuss each strategy separately, but in practice many of them can be combined to further improve robustness.

One approach to prevent declining accuracies due to transient disruptions is to use neural features that are designed to be robust against these disruptions. Historical recordings and extracellular waveform characteristics can be leveraged to identify stable units for decoder training (Ganguly and Carmena, 2009; Downey et al., 2018a). However, this approach restricts bandwidth by excluding potentially useful information from recordings. An alternative solution is to use neural decoding features that are minimally susceptible to recording instabilities. Threshold crossings are vulnerable to amplitude shifts in neural recordings, while features based on spectral power may be more robust (Zhang et al., 2018; Allahgholizadeh Haghi et al., 2019). BMIs may also leverage neural manifolds (Gallego et al., 2017, 2020; Degenhart et al., 2020), low dimensional projections that capture much of the variance in neural population activity, to combat transient disruptions. Degenhart et al. stabilize neural activity by aligning manifolds across time and show that this method can counteract recording disruptions including changes in baseline firing rate and neural tuning, as well as loss of recorded units (Degenhart et al., 2020).

Another approach to build robust decoders is careful data curation and training of the decoder parameters. In a laboratory context, a decrease in BMI performance due to task-related neural modulation can be alleviated by training neural decoders under similar conditions to the use case (Wodlinger et al., 2015). However, it is impractical to train take-home systems under every possible use-case of the BMI. Instead, recent studies suggest that deliberate neural decoder training strategies and data augmentation can help make BMIs resistant to transient disruptions. Using large amounts of historical data to train neural decoders increases the likelihood that a given model will be exposed to a variety of signal disruptions. By training with datasets containing disruptions, machine learning models may be more robust to similar disruptions that occur in the future (Sussillo et al., 2016; Schwemmer et al., 2018; Skomrock et al., 2018). Training data can also be artificially enhanced by simulating perturbations in neural decoding features that are representative of transient disruptions (Sussillo et al., 2016). These approaches may improve decoder robustness not only to recording instabilities such as array micromotion, but also to neural variability across cognitive and behavioral contexts.

**Figure 10:**
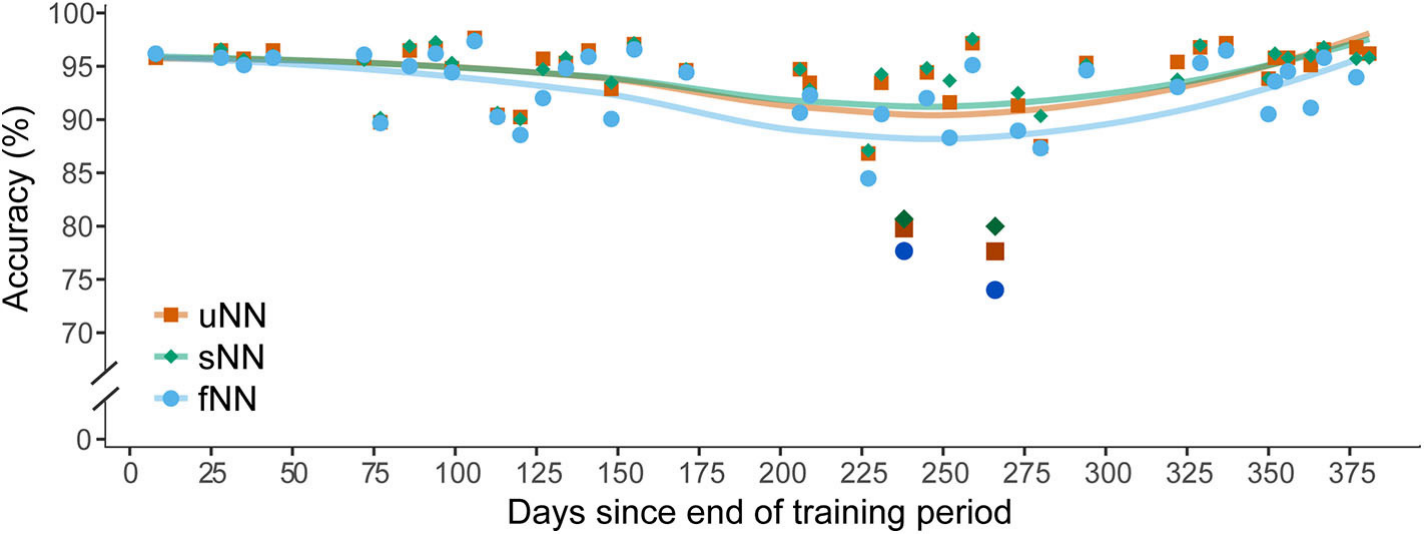
Recording disruptions impair motor intention decoding in a BMI clinical trial. The performance of three deep neural network decoder variants were evaluated for a four-movement motor imagery task over the span of 1 year. Accuracies are plotted as a function of the number of days since the end of the neural network training period. Lines denote a LOESS smoothing curve to visualize the data trends. The fixed neural network (fNN, cyan circles) decoder parameters remained unchanged for the duration of the evaluation. The other networks were updated each session with data from a preceding recording block with either explicit training labels (sNN, green diamonds) or with labels predicted by the decoder (uNN, orange squares). Both the sNN and uNN can adapt to daily changes in recording conditions and thus outperform the fNN. On days 238 and 266 (darkened data points) a disruption caused a substantial drop in accuracy for all decoding models. These dates corresponded to times when the participant had undiagnosed infections. Though the adaptive decoders are better able to compensate for this disruption, additional algorithmic interventions are needed to prevent sharp declines in decoding accuracy. Figure adapted with permission from Schwemmer et al. (2018). Copyright 2018, Springer Nature.

A third strategy is to use adaptive decoding models that combat signal instabilities through recurring parameter updates (Li et al., 2011; Bishop et al., 2014; Schwemmer et al., 2018). Adaptive decoders outperform their counterparts with fixed parameters because they account for ongoing disruptions in neural recordings. For certain BMI applications such as virtual typing, user intention can be inferred retrospectively and used to facilitate updates (Jarosiewicz et al., 2015). Recalibration methods may also use recent neuronal activity and decoder predictions obtained during BMI use to update the model, circumventing the requirement for explicit training labels (Li et al., 2011; Schwemmer et al., 2018). These self-recalibrating procedures eliminate the need for daily retraining and therefore minimize BMI setup time. Despite the remarkable performance of adaptive decoding algorithms over chronic periods, in rare cases, disruptions can still cause neural decoding accuracy to vary by up to 20% (Figure 10; Schwemmer et al., 2018). These adaptive machine learning methods may be enhanced with the decoder training strategies and neural features previously discussed.

Lastly, some transient disruptions, including electrical artifacts, may be mitigated by careful selection of referencing techniques and data filters (Paralikar et al., 2009; Young et al., 2018; Weiss et al., 2019). Common average referencing aims to remove noise and artifacts common to all electrodes by re-referencing recordings to the average potential across channels (Ludwig et al., 2009; Paralikar et al., 2009). In clinical BMI systems, a subset of electrodes with the lowest root-mean-square values are often used to calculate this reference (Jarosiewicz et al., 2015; Brandman et al., 2018). Though common average referencing can improve SNR (Ludwig et al., 2009), it can be inadequate for certain artifact removal applications because it assumes noise is similar across all electrodes (Young et al., 2018). For removing FES artifact, Young et al. propose a channel-specific referencing method and show that it outperforms common average referencing and artifact blanking (Young et al., 2018). In addition, they demonstrate neural information can be recovered during FES stimulation periods, even when the artifact is orders of magnitude larger (Young et al., 2018). Signal quality may be further enhanced by optimizing data filters. A recent study suggests that high-order filters that produce oscillatory artifacts in recordings can decrease BMI decoding accuracy (Masse et al., 2014). Furthermore, the authors show that non-causal bandpass filters can yield greater spike amplitudes and improved decoding accuracy compared to their equivalent causal filters (Masse et al., 2014). Synergistic approaches that combine multiple signal processing and decoding methods hold promise in effectively suppressing transient signal disruptions.

### Algorithmic Strategies for Reparable Disruptions

Though there are limited reports of reparable disruptions occurring with intracortical systems, we suspect these will become more prevalent as BMIs become portable. Recently and for the first time in the U.S., a portable intracortical BMI was successfully deployed in a home setting (Weiss et al., 2020). Moving outside of the lab means that system set up may be performed by caregivers instead of trained technicians, increasing the likelihood of faulty hardware connections or errors during neural decoder training. Poor connection to the percutaneous pedestal causes recording inconsistencies, reduces total available neural information, and increases the risk of irreversible damage to stimulating microelectrodes and surrounding tissue (Simeral et al., 2011; Hughes et al., 2020). In order to support BMI use outside the lab and without a technician, automated algorithms must quickly identify such malfunctions, or they may otherwise go undetected for substantial periods without technicians regularly checking signal quality.

### Algorithmic Strategies for Irreversible Compensable Disruptions

Irreversible disruptions frequently affect intracortical BMIs because the neural interface and much of the associated hardware is inaccessible without surgical intervention. Consequently, biological responses or damage to the recording device may cause permanent changes in acquired signals. Though in rare cases irreversible disruptions can result in catastrophic signal loss, many of these disruptions can be compensated for algorithmically.

Most irreversible compensable disruptions contribute to chronic attenuation or loss of recording channels. These effects can devastate BMIs with vulnerable decoding methods. For instance, Ganugly et al. demonstrate that the loss of just three neurons from a stable neural ensemble could decrease online BMI accuracy by 50% (Ganguly and Carmena, 2009). It may be possible to mitigate the effects of irreversible disruptions by leveraging data augmentation techniques during decoder training. Sussillo et al. also examined the effects of electrode dropout during online BMI control, but found that artificially perturbing firing rates during decoder training made the decoder more robust to this disruption (Sussillo et al., 2016).

Similar to compensatory strategies for transient disruptions, adaptive decoding methods that down-weight the influence of permanently damaged channels should help maintain decoding accuracy in the face of irreversible disruptions. However, this strategy will become less effective with the accumulation of irreversible failures over time. If there is insufficient information in remaining channels to maintain BMI performance, algorithmic compensation becomes increasingly difficult. Interestingly, a recent study suggests that neural dynamics under the same motor behaviors are reliable across time, regardless of recording quality (Kao et al., 2017). The authors demonstrate that neural population dynamics inferred from historical recordings with high neuron counts can be leveraged to rescue neural decoding performance after severe electrode loss, thus extending functional BMI lifetime (Kao et al., 2017).

Gradual declines in signal quality due to loss of units or material degradation may also be counteracted with targeted neural decoding features. As signals attenuate with time, and it becomes difficult to attribute electrical potentials to particular neurons, BMIs may benefit from features that salvage information from subthreshold neural activity. As an example, mean wavelet power (Bouton et al., 2016) could theoretically utilize weak or distant spiking information that is sometimes considered biological noise.

## Conclusion

Many of these algorithmic strategies will be needed in concert to mitigate the vast range of potential disruptions that intracortical BMIs face. As the diversity of BMI effectors expands from computer cursors to sophisticated devices that interact with the environment, the consequences of inaccurate predictions increase. Misspelling a word is inconvenient, but the inability to accurately control a robotic arm may pose a danger to the user and others around them. Therefore, it will be even more critical to ensure that BMIs are resilient to recording disruptions. Here we have categorized many of the common signal disruptions in hopes that it can guide in the development of targeted algorithmic solutions. Creating systems that can detect and compensate for these disruptions will be an important component in the translation of BMIs from the laboratory setting to a portable assistive device.

## Author Contributions

CD, DF, and MB conceptualized the manuscript. CD organized the literature database and wrote the first draft of the manuscript. All authors contributed to manuscript revision, read, and approved the submitted version.

## Conflict of Interest

The authors declare competing interests, as they are employed by institutions that provided the facilities for this work and/or have filed associated patents. At the time of the study, CD, SC, EM, and DF were employed by Battelle Memorial Institute. CD and MB were employed by The Ohio State University.

## References

[B1] Aflalo T. Kellis S. Klaes C. Lee B. Shi Y. Pejsa K. (2015). Decoding motor imagery from the posterior parietal cortex of a tetraplegic human. Science 348, 906–910. 10.1126/science.aaa5417 25999506 PMC4896830

[B2] Aggarwal V. Mollazadeh M. Davidson A. G. Schieber M. H. Thakor N. V. (2013). State-based decoding of hand and finger kinematics using neuronal ensemble and LFP activity during dexterous reach-to-grasp movements. J. Neurophysiol. 109, 3067–3081. 10.1152/jn.01038.2011 23536714 PMC3680811

[B3] Ajiboye A. B. Willett F. R. Young D. R. Memberg W. D. Murphy B. A. Miller J. P.. (2017). Restoration of reaching and grasping movements through brain-controlled muscle stimulation in a person with tetraplegia: a proof-of-concept demonstration. Lancet 389, 1821–1830. 10.1016/S0140-6736(17)30601-3 28363483 PMC5516547

[B4] Allahgholizadeh Haghi B. Kellis S. Shah S. Ashok M. Bashford L. Kramer D. (2019). Deep multi-state dynamic recurrent neural networks operating on wavelet based neural features for robust brain machine interfaces, in Advances in Neural Information Processing Systems 32, eds H. Wallach, H. Larochelle, A. Beygelzimer, F. Alché-Buc, E. Fox, and R. Garnett (Vancouver, BC: Curran Associates, Inc.), 14514–14525. Available online at: http://papers.nips.cc/paper/9594-deep-multi-state-dynamic-recurrent-neural-networks-operating-on-wavelet-based-neural-features-for-robust-brain-machine-interfaces.pdf (accessed April 26, 2020).

[B5] Angotzi G. N. Boi F. Lecomte A. Miele E. Malerba M. Zucca S.. (2019). SiNAPS: An implantable active pixel sensor CMOS-probe for simultaneous large-scale neural recordings. Biosens. Bioelectron. 126, 355–364. 10.1016/j.bios.2018.10.032 30466053

[B6] Barrese J. C. Aceros J. Donoghue J. P. (2016). Scanning electron microscopy of chronically implanted intracortical microelectrode arrays in non-human primates. J. Neural Eng. 13:026003. 10.1088/1741-2560/13/2/026003 26824680 PMC4854331

[B7] Barrese J. C. Rao N. Paroo K. Triebwasser C. Vargas-Irwin C. Franquemont L. (2013). Failure mode analysis of silicon-based intracortical microelectrode arrays in non-human primates. J. Neural Eng. 10:066014 10.1088/1741-2560/10/6/066014 24216311 PMC4868924

[B8] Bennett C. Mohammed F. Álvarez-Ciara A. Nguyen M. A. Dietrich W. D. Rajguru S. M.. (2019). Neuroinflammation, oxidative stress, and blood-brain barrier (BBB) disruption in acute Utah electrode array implants and the effect of deferoxamine as an iron chelator on acute foreign body response. Biomaterials 188, 144–159. 10.1016/j.biomaterials.2018.09.040 30343257 PMC6300159

[B9] Biran R. Martin D. C. Tresco P. A. (2005). Neuronal cell loss accompanies the brain tissue response to chronically implanted silicon microelectrode arrays. Exp. Neurol. 195, 115–126. 10.1016/j.expneurol.2005.04.020 16045910

[B10] Biran R. Martin D. C. Tresco P. A. (2007). The brain tissue response to implanted silicon microelectrode arrays is increased when the device is tethered to the skull. J. Biomed. Mater. Res. Part A 82A, 169–178. 10.1002/jbm.a.31138 17266019

[B11] Bishop W. Chestek C. C. Gilja V. Nuyujukian P. Foster J. D. Ryu S. I. (2014). Self-recalibrating classifiers for intracortical brain–computer interfaces. J. Neural Eng. 11:026001 10.1088/1741-2560/11/2/026001 24503597 PMC4393645

[B12] Bjornsson C. S. Oh S. J. Al-Kofahi Y. A. Lim Y. J. Smith K. L. Turner J. N.. (2006). Effects of insertion conditions on tissue strain and vascular damage during neuroprosthetic device insertion. J. Neural Eng. 3, 196–207. 10.1088/1741-2560/3/3/002 16921203

[B13] Black B. J. Kanneganti A. Joshi-Imre A. Rihani R. Chakraborty B. Abbott J. (2018). Chronic recording and electrochemical performance of Utah microelectrode arrays implanted in rat motor cortex. J. Neurophysiol. 120, 2083–2090. 10.1152/jn.00181.2018 30020844

[B14] Bockbrader M. (2019). Upper limb sensorimotor restoration through brain–computer interface technology in tetraparesis. Curr. Opin. Biomed. Eng. 11, 85–101. 10.1016/j.cobme.2019.09.002

[B15] Bockbrader M. Annetta N. Friedenberg D. Schwemmer M. Skomrock N. Colachis S. (2019). Clinically significant gains in skillful grasp coordination by an individual with tetraplegia using an implanted brain-computer interface with forearm transcutaneous muscle stimulation. Arch. Phys. Med. Rehabil. 100, 1201–1217. 10.1016/j.apmr.2018.07.445 30902630

[B16] Boehler C. Oberueber F. Schlabach S. Stieglitz T. Asplund M. (2017). Long-term stable adhesion for conducting polymers in biomedical applications: irox and nanostructured platinum solve the chronic challenge. ACS Appl. Mater. Interfaces 9, 189–197. 10.1021/acsami.6b13468 27936546

[B17] Bouton C. E. Shaikhouni A. Annetta N. V. Bockbrader M. A. Friedenberg D. A. Nielson D. M. (2016). Restoring cortical control of functional movement in a human with quadriplegia. Nature 533, 247–250. 10.1038/nature17435 27074513

[B18] Brandman D. M. Hosman T. Saab J. Burkhart M. C. Shanahan B. E. Ciancibello J. G.. (2018). Rapid calibration of an intracortical brain–computer interface for people with tetraplegia. J. Neural Eng. 15:026007. 10.1088/1741-2552/aa9ee7 29363625 PMC5823702

[B19] Bullard A. J. Hutchison B. C. Lee J. Chestek C. A. Patil P. G. (2020). Estimating risk for future intracranial, fully implanted, modular neuroprosthetic systems: a systematic review of hardware complications in clinical deep brain stimulation and experimental human intracortical arrays. Neuromodulation Technol. Neural Interface 23, 411–426. 10.1111/ner.13069 31747103

[B20] Buzsáki G. (2004). Large-scale recording of neuronal ensembles. Nat. Neurosci. 7, 446–451. 10.1038/nn1233 15114356

[B21] Caldwell R. Sharma R. Takmakov P. Street M. G. Solzbacher F. Tathireddy P. (2018). Neural electrode resilience against dielectric damage may be improved by use of highly doped silicon as a conductive material. J. Neurosci. Methods 293, 210–225. 10.1016/j.jneumeth.2017.10.002 29017900

[B22] Campbell A. Wu C. (2018). Chronically implanted intracranial electrodes: tissue reaction and electrical changes. Micromachines 9:430. 10.3390/mi9090430 30424363 PMC6187588

[B23] Campbell P. K. Jones K. E. Huber R. J. Horch K. W. Normann R. A. (1991). A silicon-based, three-dimensional neural interface: manufacturing processes for an intracortical electrode array. IEEE Trans. Biomed. Eng. 38, 758–768. 10.1109/10.83588 1937509

[B24] Cody P. A. Eles J. R. Lagenaur C. F. Kozai T. D. Y. Cui X. T. (2018). Unique electrophysiological and impedance signatures between encapsulation types: An analysis of biological Utah array failure and benefit of a biomimetic coating in a rat model. Biomaterials 161, 117–128. 10.1016/j.biomaterials.2018.01.025 29421549 PMC5817007

[B25] Cogan S. F. (2008). Neural stimulation and recording electrodes. Annu. Rev. Biomed. Eng. 10, 275–309. 10.1146/annurev.bioeng.10.061807.160518 18429704

[B26] Cogan S. F. Guzelian A. A. Agnew W. F. Yuen T. G. McCreery D. B. (2004). Over-pulsing degrades activated iridium oxide films used for intracortical neural stimulation. J. Neurosci. Methods 137, 141–150. 10.1016/j.jneumeth.2004.02.019 15262054

[B27] Colachis S. C. Bockbrader M. A. Zhang M. Friedenberg D. A. Annetta N. V. Schwemmer M. A. (2018). Dexterous control of seven functional hand movements using cortically-controlled transcutaneous muscle stimulation in a person with tetraplegia. Front. Neurosci. 12, 1–14. 10.3389/fnins.2018.00208 29670506 PMC5893794

[B28] Collinger J. L. Boninger M. L. Bruns T. M. Curley K. Wang W. Weber D. J. (2013a). Functional priorities, assistive technology, and brain-computer interfaces after spinal cord injury. J. Rehabil. Res. Dev. 50:145 10.1682/JRRD.2011.11.0213 23760996 PMC3684986

[B29] Collinger J. L. Wodlinger B. Downey J. E. Wang W. Tyler-Kabara E. C. Weber D. J. (2013b). High-performance neuroprosthetic control by an individual with tetraplegia. Lancet 381, 557–564. 10.1016/S0140-6736(12)61816-9 23253623 PMC3641862

[B30] Cui X. T. Zhou D. D. (2007). Poly (3,4-Ethylenedioxythiophene) for Chronic Neural Stimulation. IEEE Trans. Neural Syst. Rehabil. Eng. 15, 502–508. 10.1109/TNSRE.2007.909811 18198707

[B31] Dantzer R. O'Connor J. C. Freund G. G. Johnson R. W. Kelley K. W. (2008). From inflammation to sickness and depression: when the immune system subjugates the brain. Nat. Rev. Neurosci. 9, 46–56. 10.1038/nrn2297 18073775 PMC2919277

[B32] Degenhart A. D. Bishop W. E. Oby E. R. Tyler-Kabara E. C. Chase S. M. Batista A. P.. (2020). Stabilization of a brain–computer interface via the alignment of low-dimensional spaces of neural activity. Nat. Biomed. Eng. 4, 672–685. 10.1038/s41551-020-0542-9 32313100 PMC7822646

[B33] Dickey A. S. Suminski A. Amit Y. Hatsopoulos N. G. (2009). Single-unit stability using chronically implanted multielectrode arrays. J. Neurophysiol. 102, 1331–1339. 10.1152/jn.90920.2008 19535480 PMC2724357

[B34] Dobkin B. H. (2007). Brain-computer interface technology as a tool to augment plasticity and outcomes for neurological rehabilitation. J. Physiol. 579, 637–642. 10.1113/jphysiol.2006.123067 17095557 PMC2151380

[B35] Downey J. E. Brane L. Gaunt R. A. Tyler-Kabara E. C. Boninger M. L. Collinger J. L. (2017). Motor cortical activity changes during neuroprosthetic-controlled object interaction. Sci. Rep. 7:16947 10.1038/s41598-017-17222-3 29209023 PMC5717217

[B36] Downey J. E. Schwed N. Chase S. M. Schwartz A. B. Collinger J. L. (2018a). Intracortical recording stability in human brain–computer interface users. J. Neural Eng. 15:046016 10.1088/1741-2552/aab7a0 29553484

[B37] Downey J. E. Weiss J. M. Flesher S. N. Thumser Z. C. Marasco P. D. Boninger M. L.. (2018b). Implicit grasp force representation in human motor cortical recordings. Front. Neurosci. 12, 1–7. 10.3389/fnins.2018.00801 30429772 PMC6220062

[B38] Du Z. J. Kolarcik C. L. Kozai T. D. Y. Luebben S. D. Sapp S. A. Zheng X. S.. (2017). Ultrasoft microwire neural electrodes improve chronic tissue integration. Acta Biomater. 53, 46–58. 10.1016/j.actbio.2017.02.010 28185910 PMC5512864

[B39] Eles J. R. Vazquez A. L. Kozai T. D. Y. Cui X. T. (2019). Meningeal inflammatory response and fibrous tissue remodeling around intracortical implants: An *in vivo* two-photon imaging study. Biomaterials 195, 111–123. 10.1016/j.biomaterials.2018.12.031 30634095 PMC6350934

[B40] Ereifej E. S. Rial G. M. Hermann J. K. Smith C. S. Meade S. M. Rayyan J. M. (2018). Implantation of neural probes in the brain elicits oxidative stress. Front. Bioeng. Biotechnol. 6:9 10.3389/fbioe.2018.00009 29487848 PMC5816578

[B41] Fagg A. H. Ojakangas G. W. Miller L. E. Hatsopoulos N. G. (2009). Kinetic trajectory decoding using motor cortical ensembles. IEEE Trans. Neural Syst. Rehabil. Eng. 17, 487–496. 10.1109/TNSRE.2009.2029313 19666343

[B42] Fang C. Wong T.-M. Lau T.-W. To K. K. W. Wong S. S. Y. Leung F. (2017). Infection after fracture osteosynthesis – Part I. J. Orthop. Surg. 25:230949901769271 10.1177/2309499017692712 28215118

[B43] Fawcett J. W. Asher R. (1999). The glial scar and central nervous system repair. Brain Res. Bull. 49, 377–391. 10.1016/S0361-9230(99)00072-6 10483914

[B44] Fernández E. Greger B. House P. A. Aranda I. Botella C. Albisua J.. (2014). Acute human brain responses to intracortical microelectrode arrays: challenges and future prospects. Front. Neuroeng. 7, 1–6. 10.3389/fneng.2014.00024 25100989 PMC4104831

[B45] Fiáth R. Raducanu B. C. Musa S. Andrei A. Lopez C. M. van Hoof C. (2018). A silicon-based neural probe with densely-packed low-impedance titanium nitride microelectrodes for ultrahigh-resolution *in vivo* recordings. Biosens. Bioelectron. 106, 86–92. 10.1016/j.bios.2018.01.060 29414094

[B46] Flesher S. Downey J. Collinger J. Foldes S. Weiss J. Tyler-Kabara E. (2017). Intracortical microstimulation as a feedback source for brain-computer interface users, in Brain-Computer Interface Research: A State-of-the-Art Summary 6, eds Guger C. Allison B. Lebedev M. (Cham: Springer International Publishing), 43–54. 10.1007/978-3-319-64373-1_5

[B47] Flesher S. N. Collinger J. L. Foldes S. T. Weiss J. M. Downey J. E. Tyler-Kabara E. C.. (2016). Intracortical microstimulation of human somatosensory cortex. Sci. Transl. Med. 8, 1–11. 10.1126/scitranslmed.aaf8083 27738096

[B48] Flint R. D. Scheid M. R. Wright Z. A. Solla S. A. Slutzky M. W. (2016). Long-term stability of motor cortical activity: implications for brain machine interfaces and optimal feedback control. J. Neurosci. 36, 3623–3632. 10.1523/JNEUROSCI.2339-15.2016 27013690 PMC4804017

[B49] Frere S. Slutsky I. (2018). Alzheimer's disease: from firing instability to homeostasis network collapse. Neuron 97, 32–58. 10.1016/j.neuron.2017.11.028 29301104

[B50] Gaire J. Lee H. C. Hilborn N. Ward R. Regan M. Otto K. J. (2018). The role of inflammation on the functionality of intracortical microelectrodes. J. Neural Eng. 15:066027. 10.1088/1741-2552/aae4b6 30260321

[B51] Gallego J. A. Perich M. G. Chowdhury R. H. Solla S. A. Miller L. E. (2020). Long-term stability of cortical population dynamics underlying consistent behavior. Nat. Neurosci. 23, 260–270. 10.1038/s41593-019-0555-4 31907438 PMC7007364

[B52] Gallego J. A. Perich M. G. Miller L. E. Solla S. A. (2017). Neural manifolds for the control of movement. Neuron 94, 978–984. 10.1016/j.neuron.2017.05.025 28595054 PMC6122849

[B53] Ganguly K. Carmena J. M. (2009). Emergence of a stable cortical map for neuroprosthetic control. PLoS Biol. 7:e1000153. 10.1371/journal.pbio.1000153 19621062 PMC2702684

[B54] Ganzer P. D. Colachis S. C. Schwemmer M. A. Friedenberg D. A. Dunlap C. F. Swiftney C. E. (2020). Restoring the sense of touch using a sensorimotor demultiplexing neural interface. Cell 181, 763–773.e12. 10.1016/j.cell.2020.03.054 32330415

[B55] Garcia-Arguello L. Y. O'Horo J. C. Farrell A. Blakney R. Sohail M. R. Evans C. T. (2017). Infections in the spinal cord-injured population: a systematic review. Spinal Cord 55, 526–534. 10.1038/sc.2016.173 27922625

[B56] Gilgunn P. J. Ong X. C. Flesher S. N. Schwartz A. B. Gaunt R. A. (2013). Structural analysis of explanted microelectrode arrays, in 2013 6th International IEEE/EMBS Conference on Neural Engineering (NER) (IEEE), 719–722. 10.1109/NER.2013.6696035

[B57] Gwon T. M. Kim J. H. Choi G. J. Kim S. J. (2016). Mechanical interlocking to improve metal–polymer adhesion in polymer-based neural electrodes and its impact on device reliability. J. Mater. Sci. 51, 6897–6912. 10.1007/s10853-016-9977-5

[B58] Hassibi A. Navid R. Dutton R. W. Lee T. H. (2004). Comprehensive study of noise processes in electrode electrolyte interfaces. J. Appl. Phys. 96, 1074–1082. 10.1063/1.1755429

[B59] Hochberg L. R. Bacher D. Jarosiewicz B. Masse N. Y. Simeral J. D. Vogel J.. (2012). Reach and grasp by people with tetraplegia using a neurally controlled robotic arm. Nature 485, 372–375. 10.1038/nature11076 22596161 PMC3640850

[B60] Hochberg L. R. Donoghue J. P. (2006). Sensors for brain-computer interfaces. IEEE Eng. Med. Biol. Mag. 25, 32–38. 10.1109/MEMB.2006.1705745 17020197

[B61] Hochberg L. R. Serruya M. D. Friehs G. M. Mukand J. A. Saleh M. Caplan A. H.. (2006). Neuronal ensemble control of prosthetic devices by a human with tetraplegia. Nature 442, 164–171. 10.1038/nature04970 16838014

[B62] House P. A. MacDonald J. D. Tresco P. A. Normann R. A. (2006). Acute microelectrode array implantation into human neocortex: preliminary technique and histological considerations. Neurosurg. Focus 20, 1–4. 10.3171/foc.2006.20.5.5 16711661

[B63] Hsu J.-M. Rieth L. Normann R. A. Tathireddy P. Solzbacher F. (2009). Encapsulation of an integrated neural interface device with parylene C. IEEE Trans. Biomed. Eng. 56, 23–29. 10.1109/TBME.2008.2002155 19224715

[B64] Hughes C. L. Flesher S. N. Weiss J. M. Downey J. E. Collinger J. L. Gaunt R. A. (2020). Neural stimulation and recording performance in human somatosensory cortex over 1500 days. medRxiv [Preprint]. 10.1101/2020.01.21.20018341 PMC8500669 34320481

[B65] Ising C. Venegas C. Zhang S. Scheiblich H. Schmidt S. V. Vieira-Saecker A.. (2019). NLRP3 inflammasome activation drives tau pathology. Nature 575, 669–673. 10.1038/s41586-019-1769-z 31748742 PMC7324015

[B66] Jarosiewicz B. Sarma A. A. Bacher D. Masse N. Y. Simeral J. D. Sorice B. (2015). Virtual typing by people with tetraplegia using a self-calibrating intracortical brain-computer interface. Sci. Transl. Med. 7:313ra179. 10.1126/scitranslmed.aac7328 PMC4765319 26560357

[B67] Jorfi M. Skousen J. L. Weder C. Capadona J. R. (2015). Progress towards biocompatible intracortical microelectrodes for neural interfacing applications. J. Neural Eng. 12:011001 10.1088/1741-2560/12/1/011001 25460808 PMC4428498

[B68] Jun J. J. Steinmetz N. A. Siegle J. H. Denman D. J. Bauza M. Barbarits B.. (2017). Fully integrated silicon probes for high-density recording of neural activity. Nature 551, 232–236. 10.1038/nature24636 29120427 PMC5955206

[B69] Kane S. R. Cogan S. F. Ehrlich J. Plante T. D. McCreery D. B. Troyk P. R. (2013). Electrical performance of penetrating microelectrodes chronically implanted in cat cortex. IEEE Trans. Biomed. Eng. 60, 2153–2160. 10.1109/TBME.2013.2248152 23475329 PMC7441534

[B70] Kao J. C. Ryu S. I. Shenoy K. V. (2017). Leveraging neural dynamics to extend functional lifetime of brain-machine interfaces. Sci. Rep. 7:7395. 10.1038/s41598-017-06029-x 28784984 PMC5547077

[B71] Karumbaiah L. Saxena T. Carlson D. Patil K. Patkar R. Gaupp E. A.. (2013). Relationship between intracortical electrode design and chronic recording function. Biomaterials 34, 8061–8074. 10.1016/j.biomaterials.2013.07.016 23891081

[B72] Kennedy P. (2011). Changes in emotional state modulate neuronal firing rates of human speech motor cortex: A case study in long-term recording. Neurocase 17, 381–393. 10.1080/13554794.2010.532137 21967282 PMC3187572

[B73] Klaes C. (2018). Invasive brain-computer interfaces and neural recordings from humans, in Handbook of in vivo Neural Plasticity Techniques Handbook of Behavioral Neuroscience, ed Manahan-Vaughan D. (London: Elsevier), 527–539. 10.1016/B978-0-12-812028-6.00028-8

[B74] Klaes C. Kellis S. Aflalo T. Lee B. Pejsa K. Shanfield K.. (2015). Hand shape representations in the human posterior parietal cortex. J. Neurosci. 35, 15466–15476. 10.1523/JNEUROSCI.2747-15.2015 26586832 PMC4649012

[B75] Kozai T. D. Y. Catt K. Li X. Gugel Z. V. Olafsson V. T. Vazquez A. L.. (2015a). Mechanical failure modes of chronically implanted planar silicon-based neural probes for laminar recording. Biomaterials 37, 25–39. 10.1016/j.biomaterials.2014.10.040 25453935 PMC4312222

[B76] Kozai T. D. Y. Jaquins-Gerstl A. S. Vazquez A. L. Michael A. C. Cui X. T. (2015b). Brain tissue responses to neural implants impact signal sensitivity and intervention strategies. ACS Chem. Neurosci. 6, 48–67. 10.1021/cn500256e 25546652 PMC4304489

[B77] Kozai T. D. Y. Vazquez A. L. Weaver C. L. Kim S.-G. Cui X. T. (2012). *In vivo* two-photon microscopy reveals immediate microglial reaction to implantation of microelectrode through extension of processes. J. Neural Eng. 9:066001. 10.1088/1741-2560/9/6/066001 23075490 PMC3511663

[B78] Krüger J. Caruana F. Volta R. D. Rizzolatti G. (2010). Seven years of recording from monkey cortex with a chronically implanted multiple microelectrode. Front. Neuroeng. 3:6 10.3389/fneng.2010.00006 20577628 PMC2889715

[B79] Lebedev M. A. Nicolelis M. A. L. (2006). Brain–machine interfaces: past, present and future. Trends Neurosci. 29, 536–546. 10.1016/j.tins.2006.07.004 16859758

[B80] Leber M. Körner J. Reiche C. F. Yin M. Bhandari R. Franklin R.. (2019). Advances in penetrating multichannel microelectrodes based on the utah array platform, in Neural Interface: Frontiers and Applications, ed Zheng X. (Singapore: Springer Singapore), 1–40. 10.1007/978-981-13-2050-7_1 31729670

[B81] Lecomte A. Descamps E. Bergaud C. (2018). A review on mechanical considerations for chronically-implanted neural probes. J. Neural Eng. 15:031001. 10.1088/1741-2552/aa8b4f 28885187

[B82] Lempka S. F. Johnson M. D. Moffitt M. A. Otto K. J. Kipke D. R. McIntyre C. C. (2011). Theoretical analysis of intracortical microelectrode recordings. J. Neural Eng. 8:045006. 10.1088/1741-2560/8/4/045006 21775783 PMC3196618

[B83] Li Z. O'Doherty J. E. Lebedev M. A. Nicolelis M. A. L. (2011). Adaptive decoding for brain-machine interfaces through bayesian parameter updates. Neural Comput. 23, 3162–3204. 10.1162/NECO_a_00207 21919788 PMC3335277

[B84] Lopez C. M. Welkenhuysen M. Musa S. Eberle W. Bartic C. Puers R. (2012). Towards a noise prediction model for *in vivo* neural recording, in 2012 Annual International Conference of the IEEE Engineering in Medicine and Biology Society (San Diego, CA: IEEE), 759–762. 10.1109/EMBC.2012.6346042 23366003

[B85] Ludwig K. A. Miriani R. M. Langhals N. B. Joseph M. D. Anderson D. J. Kipke D. R. (2009). Using a common average reference to improve cortical neuron recordings from microelectrode arrays. J. Neurophysiol. 101, 1679–1689. 10.1152/jn.90989.2008 19109453 PMC2666412

[B86] Malaga K. A. Schroeder K. E. Patel P. R. Irwin Z. T. Thompson D. E. Nicole Bentley J.. (2016). Data-driven model comparing the effects of glial scarring and interface interactions on chronic neural recordings in non-human primates. J. Neural Eng. 13:016010. 10.1088/1741-2560/13/1/016010 26655972

[B87] Masse N. Y. Jarosiewicz B. Simeral J. D. Bacher D. Stavisky S. D. Cash S. S.. (2014). Non-causal spike filtering improves decoding of movement intention for intracortical BCIs. J. Neurosci. Methods 236, 58–67. 10.1016/j.jneumeth.2014.08.004 25128256 PMC4169749

[B88] McConnell G. C. Butera R. J. Bellamkonda R. V. (2009a). Bioimpedance modeling to monitor astrocytic response to chronically implanted electrodes. J. Neural Eng. 6:055005. 10.1088/1741-2560/6/5/055005 19721187

[B89] McConnell G. C. Rees H. D. Levey A. I. Gutekunst C.-A. Gross R. E. Bellamkonda R. V. (2009b). Implanted neural electrodes cause chronic, local inflammation that is correlated with local neurodegeneration. J. Neural Eng. 6:056003. 10.1088/1741-2560/6/5/056003 19700815

[B90] Mckee A. C. Daneshvar D. H. (2015). The neuropathology of traumatic brain injury, in Handbook of Clinical Neurology, eds Grafman J. Salazar A. M. (Amsterdam: Elsevier), 45–66. 10.1016/B978-0-444-52892-6.00004-0 PMC4694720 25702209

[B91] McMorland A. J. C. Velliste M. (2013). Baseplate for two-stage cranial mounting of BMI connectors. J. Neural Eng. 10:034001 10.1088/1741-2560/10/3/034001 23594571

[B92] Mercanzini A. Colin P. Bensadoun J.-C. Bertsch A. Renaud P. (2009). *In vivo* Electrical impedance spectroscopy of tissue reaction to microelectrode arrays. IEEE Trans. Biomed. Eng. 56, 1909–1918. 10.1109/TBME.2009.2018457 19362904

[B93] Michelson N. J. Vazquez A. L. Eles J. R. Salatino J. W. Purcell E. K. Williams J. J.. (2018). Multi-scale, multi-modal analysis uncovers complex relationship at the brain tissue-implant neural interface: new emphasis on the biological interface. J. Neural Eng. 15:033001. 10.1088/1741-2552/aa9dae 29182149 PMC5967409

[B94] Moffitt M. A. McIntyre C. C. (2005). Model-based analysis of cortical recording with silicon microelectrodes. Clin. Neurophysiol. 116, 2240–2250. 10.1016/j.clinph.2005.05.018 16055377

[B95] Nguyen J. K. Park D. J. Skousen J. L. Hess-Dunning A. E. Tyler D. J. Rowan S. J.. (2014). Mechanically-compliant intracortical implants reduce the neuroinflammatory response. J. Neural Eng. 11:056014. 10.1088/1741-2560/11/5/056014 25125443 PMC4175058

[B96] Nicolas-Alonso L. F. Gomez-Gil J. (2012). Brain computer interfaces, a review. Sensors 12, 1211–1279. 10.3390/s120201211 22438708 PMC3304110

[B97] Nicolelis M. A. L. Dimitrov D. Carmena J. M. Crist R. Lehew G. Kralik J. D. (2003). Chronic, multisite, multielectrode recordings in macaque monkeys. Proc. Natl. Acad. Sci U.S.A. 100, 11041–11046. 10.1073/pnas.1934665100 12960378 PMC196923

[B98] Nolta N. F. Christensen M. B. Crane P. D. Skousen J. L. Tresco P. A. (2015). BBB leakage, astrogliosis, and tissue loss correlate with silicon microelectrode array recording performance. Biomaterials 53, 753–762. 10.1016/j.biomaterials.2015.02.081 25890770

[B99] Obaid A. Hanna M.-E. Wu Y.-W. Kollo M. Racz R. Angle M. R. (2020). Massively parallel microwire arrays integrated with CMOS chips for neural recording. Sci. Adv. 6:eaay2789 10.1126/sciadv.aay2789 32219158 PMC7083623

[B100] Pandarinath C. Nuyujukian P. Blabe C. H. Sorice B. L. Saab J. Willett F. R. (2017). High performance communication by people with paralysis using an intracortical brain-computer interface. Elife 6, 1–27. 10.7554/eLife.18554 PMC5319839 28220753

[B101] Paralikar K. J. Rao C. R. Clement R. S. (2009). New approaches to eliminating common-noise artifacts in recordings from intracortical microelectrode arrays: Inter-electrode correlation and virtual referencing. J. Neurosci. Methods 181, 27–35. 10.1016/j.jneumeth.2009.04.014 19394363 PMC2702226

[B102] Patrick E. Orazem M. E. Sanchez J. C. Nishida T. (2011). Corrosion of tungsten microelectrodes used in neural recording applications. J. Neurosci. Methods 198, 158–171. 10.1016/j.jneumeth.2011.03.012 21470563 PMC3107924

[B103] Perge J. A. Homer M. L. Malik W. Q. Cash S. Eskandar E. Friehs G.. (2013). Intra-day signal instabilities affect decoding performance in an intracortical neural interface system. J. Neural Eng. 10:036004. 10.1088/1741-2560/10/3/036004 23574741 PMC3693851

[B104] Perge J. A. Zhang S. Malik W. Q. Homer M. L. Cash S. Friehs G. (2014). Reliability of directional information in unsorted spikes and local field potentials recorded in human motor cortex. J. Neural Eng. 11:046007 10.1088/1741-2560/11/4/046007 24921388 PMC4142142

[B105] Potter K. A. Buck A. C. Self W. K. Callanan M. E. Sunil S. Capadona J. R. (2013). The effect of resveratrol on neurodegeneration and blood brain barrier stability surrounding intracortical microelectrodes. Biomaterials 34, 7001–7015. 10.1016/j.biomaterials.2013.05.035 23791503

[B106] Potter K. A. Buck A. C. Self W. K. Capadona J. R. (2012). Stab injury and device implantation within the brain results in inversely multiphasic neuroinflammatory and neurodegenerative responses. J. Neural Eng. 9:046020. 10.1088/1741-2560/9/4/046020 22832283

[B107] Potter-Baker K. A. Capadona J. R. (2015). Reducing the “Stress”: antioxidative therapeutic and material approaches may prevent intracortical microelectrode failure. ACS Macro Lett. 4, 275–279. 10.1021/mz500743a 35596335

[B108] Prasad A. Sanchez J. C. (2012). Quantifying long-term microelectrode array functionality using chronic *in vivo* impedance testing. J. Neural Eng. 9:026028. 10.1088/1741-2560/9/2/026028 22442134

[B109] Prasad A. Xue Q.-S. Dieme R. Sankar V. Mayrand R. C. Nishida T. (2014). Abiotic-biotic characterization of Pt/Ir microelectrode arrays in chronic implants. Front. Neuroeng. 7, 1–15. 10.3389/fneng.2014.00002 24550823 PMC3912984

[B110] Prasad A. Xue Q.-S. Sankar V. Nishida T. Shaw G. Streit W. J. (2012). Comprehensive characterization and failure modes of tungsten microwire arrays in chronic neural implants. J. Neural Eng. 9:056015 10.1088/1741-2560/9/5/056015 23010756

[B111] Purcell E. K. Thompson D. E. Ludwig K. A. Kipke D. R. (2009). Flavopiridol reduces the impedance of neural prostheses *in vivo* without affecting recording quality. J. Neurosci. Methods 183, 149–157. 10.1016/j.jneumeth.2009.06.026 19560490

[B112] Putzeys J. Musa S. Mora Lopez C. Raducanu B. C. Carton A. De Ceulaer J. (2019). Neuropixels data-acquisition system: a scalable platform for parallel recording of 10 000+ electrophysiological signals. IEEE Trans. Biomed. Circuits Syst. 13, 1635–1644. 10.1109/TBCAS.2019.2943077 31545742

[B113] Ravikumar M. Hageman D. J. Tomaszewski W. H. Chandra G. M. Skousen J. L. Capadona J. R. (2014). The effect of residual endotoxin contamination on the neuroinflammatory response to sterilized intracortical microelectrodes. J. Mater. Chem. B 2, 2517–2529. 10.1039/C3TB21453B 24778808 PMC4000032

[B114] Roitbak T. Syková E. (1999). Diffusion barriers evoked in the rat cortex by reactive astrogliosis. Glia 28, 40–48. 10.1002/(SICI)1098-1136(199910)28:1<40::AID-GLIA5>3.0.CO 10498821

[B115] Rousche P. J. Normann R. A. (1998). Chronic recording capability of the utah intracortical electrode array in cat sensory cortex. J. Neurosci. Methods 82, 1–15. 10.1016/S0165-0270(98)00031-4 10223510

[B116] Salatino J. W. Ludwig K. A. Kozai T. D. Y. Purcell E. K. (2017). Glial responses to implanted electrodes in the brain. Nat. Biomed. Eng. 1, 862–877. 10.1038/s41551-017-0154-1 30505625 PMC6261524

[B117] Sanes J. N. Donoghue J. P. (2000). Plasticity and primary motor cortex. Annu. Rev. Neurosci. 23, 393–415. 10.1146/annurev.neuro.23.1.393 10845069

[B118] Santhanam G. Linderman M. D. Gilja V. Afshar A. Ryu S. I. Meng T. H.. (2007). HermesB: a continuous neural recording system for freely behaving primates. IEEE Trans. Biomed. Eng. 54, 2037–2050. 10.1109/TBME.2007.895753 18018699

[B119] Saxena T. Karumbaiah L. Gaupp E. A. Patkar R. Patil K. Betancur M.. (2013). The impact of chronic blood–brain barrier breach on intracortical electrode function. Biomaterials 34, 4703–4713. 10.1016/j.biomaterials.2013.03.007 23562053

[B120] Schmitt G. Schultze J.-W. Faßbender F. Bu,ß G. Lüth H. Schöning M. J. (1999). Passivation and corrosion of microelectrode arrays. Electrochim. Acta 44, 3865–3883. 10.1016/S0013-4686(99)00094-8

[B121] Schwarz D. A. Lebedev M. A. Hanson T. L. Dimitrov D. F. Lehew G. Meloy J.. (2014). Chronic, wireless recordings of large-scale brain activity in freely moving rhesus monkeys. Nat. Methods 11, 670–676. 10.1038/nmeth.2936 24776634 PMC4161037

[B122] Schwemmer M. A. Skomrock N. D. Sederberg P. B. Ting J. E. Sharma G. Bockbrader M. A.. (2018). Meeting brain–computer interface user performance expectations using a deep neural network decoding framework. Nat. Med. 24, 1669–1676. 10.1038/s41591-018-0171-y 30250141

[B123] Scott S. H. Kalaska J. F. (1995). Changes in motor cortex activity during reaching movements with similar hand paths but different arm postures. J. Neurophysiol. 73, 2563–2567. 10.1152/jn.1995.73.6.2563 7666162

[B124] Seymour J. P. Elkasabi Y. M. Chen H.-Y. Lahann J. Kipke D. R. (2009). The insulation performance of reactive parylene films in implantable electronic devices. Biomaterials 30, 6158–6167. 10.1016/j.biomaterials.2009.07.061 19703712 PMC2748105

[B125] Shenoy K. V. Carmena J. M. (2014). Combining decoder design and neural adaptation in brain-machine interfaces. Neuron 84, 665–680. 10.1016/j.neuron.2014.08.038 25459407

[B126] Simeral J. D. Kim S.-P. Black M. J. Donoghue J. P. Hochberg L. R. (2011). Neural control of cursor trajectory and click by a human with tetraplegia 1000 days after implant of an intracortical microelectrode array. J. Neural Eng. 8:025027 10.1088/1741-2560/8/2/025027 21436513 PMC3715131

[B127] Šišková Z. Justus D. Kaneko H. Friedrichs D. Henneberg N. Beutel T.. (2014). Dendritic structural degeneration is functionally linked to cellular hyperexcitability in a mouse model of alzheimer's disease. Neuron 84, 1023–1033. 10.1016/j.neuron.2014.10.024 25456500

[B128] Skomrock N. D. Schwemmer M. A. Ting J. E. Trivedi H. R. Sharma G. Bockbrader M. A.. (2018). A characterization of brain-computer interface performance trade-offs using support vector machines and deep neural networks to decode movement intent. Front. Neurosci. 12:763. 10.3389/fnins.2018.00763 30459542 PMC6232881

[B129] Sridharan A. Nguyen J. K. Capadona J. R. Muthuswamy J. (2015). Compliant intracortical implants reduce strains and strain rates in brain tissue *in vivo*. J. Neural Eng. 12:036002. 10.1088/1741-2560/12/3/036002 25834105 PMC4460006

[B130] Steinmetz P. N. Roy A. Fitzgerald P. J. Hsiao S. S. Johnson K. O. Niebur E. (2000). Attention modulates synchronized neuronal firing in primate somatosensory cortex. Nature 404, 187–190. 10.1038/35004588 10724171

[B131] Straka M. Shafer B. Vasudevan S. Welle C. Rieth L. (2018). Characterizing longitudinal changes in the impedance spectra of in-vivo peripheral nerve electrodes. Micromachines 9:587. 10.3390/mi9110587 30424513 PMC6266965

[B132] Subbaroyan J. Martin D. C. Kipke D. R. (2005). A finite-element model of the mechanical effects of implantable microelectrodes in the cerebral cortex. J. Neural Eng. 2, 103–113. 10.1088/1741-2560/2/4/006 16317234

[B133] Sussillo D. Stavisky S. D. Kao J. C. Ryu S. I. Shenoy K. V. (2016). Making brain–machine interfaces robust to future neural variability. Nat. Commun. 7:13749. 10.1038/ncomms13749 27958268 PMC5159828

[B134] Szarowski D. H. Andersen M. D. Retterer S. Spence A. J. Isaacson M. Craighead H. G.. (2003). Brain responses to micro-machined silicon devices. Brain Res. 983, 23–35. 10.1016/S0006-8993(03)03023-3 12914963

[B135] Takmakov P. Ruda K. Scott Phillips K. Isayeva I. S. Krauthamer V. Welle C. G. (2015). Rapid evaluation of the durability of cortical neural implants using accelerated aging with reactive oxygen species. J. Neural Eng. 12:026003. 10.1088/1741-2560/12/2/026003 25627426 PMC5542586

[B136] Teeling J. L. Perry V. H. (2009). Systemic infection and inflammation in acute CNS injury and chronic neurodegeneration: underlying mechanisms. Neuroscience 158, 1062–1073. 10.1016/j.neuroscience.2008.07.031 18706982

[B137] Thomas T. M. Nickl R. W. Thompson M. C. Candrea D. N. Fifer M. S. McMullen D. P. (2020). Simultaneous classification of bilateral hand gestures using bilateral microelectrode recordings in a tetraplegic patient. medRxiv [Preprint]. 10.1101/2020.06.02.20116913

[B138] Troyk P. R. Detlefsen D. E. Cogan S. F. Ehrlich J. Bak M. McCreery D. B. (2004). Safe” charge-injection waveforms for iridium oxide (AIROF) microelectrodes, in The 26th Annual International Conference of the IEEE Engineering in Medicine and Biology Society (San Francisco, CA: IEEE), 4141–4144. 10.1109/IEMBS.2004.1404155 17271213

[B139] Vargas-Irwin C. E. Shakhnarovich G. Yadollahpour P. Mislow J. M. K. Black M. J. Donoghue J. P. (2010). Decoding complete reach and grasp actions from local primary motor cortex populations. J. Neurosci. 30, 9659–9669. 10.1523/JNEUROSCI.5443-09.2010 20660249 PMC2921895

[B140] Velliste M. Kennedy S. D. Schwartz A. B. Whitford A. S. Sohn J.-W. McMorland A. J. C. (2014). Motor cortical correlates of arm resting in the context of a reaching task and implications for prosthetic control. J. Neurosci. 34, 6011–6022. 10.1523/JNEUROSCI.3520-13.2014 24760860 PMC3996218

[B141] Vezzani A. Viviani B. (2015). Neuromodulatory properties of inflammatory cytokines and their impact on neuronal excitability. Neuropharmacology 96, 70–82. 10.1016/j.neuropharm.2014.10.027 25445483

[B142] Weiss J. M. Flesher S. N. Franklin R. Collinger J. L. Gaunt R. A. (2019). Artifact-free recordings in human bidirectional brain–computer interfaces. J. Neural Eng. 16:016002. 10.1088/1741-2552/aae748 30444217

[B143] Weiss J. M. Gaunt R. A. Franklin R. Boninger M. L. Collinger J. L. (2020). Demonstration of a portable intracortical brain-computer interface. Brain-Computer Interfaces 6, 106–117. 10.1080/2326263X.2019.1709260

[B144] Wellman S. M. Eles J. R. Ludwig K. A. Seymour J. P. Michelson N. J. McFadden W. E. (2018). A materials roadmap to functional neural interface design. Adv. Funct. Mater. 28:1701269 10.1002/adfm.201701269 29805350 PMC5963731

[B145] Western Electric Company (1956). Statistical Quality Control Handbook. Indianapolis: Western Electric Company.

[B146] Williams J. C. Hippensteel J. A. Dilgen J. Shain W. Kipke D. R. (2007). Complex impedance spectroscopy for monitoring tissue responses to inserted neural implants. J. Neural Eng. 4, 410–423. 10.1088/1741-2560/4/4/007 18057508

[B147] Winslow B. D. Christensen M. B. Yang W.-K. Solzbacher F. Tresco P. A. (2010). A comparison of the tissue response to chronically implanted Parylene-C-coated and uncoated planar silicon microelectrode arrays in rat cortex. Biomaterials 31, 9163–9172. 10.1016/j.biomaterials.2010.05.050 20561678 PMC12327938

[B148] Wise K. D. (2005). Silicon microsystems for neuroscience and neural prostheses. IEEE Eng. Med. Biol. Mag. 24, 22–29. 10.1109/MEMB.2005.1511497 16248114

[B149] Wodlinger B. Downey J. E. Tyler-Kabara E. C. Schwartz A. B. Boninger M. L. Collinger J. L. (2015). Ten-dimensional anthropomorphic arm control in a human brain–machine interface: difficulties, solutions, and limitations. J. Neural Eng. 12:016011. 10.1088/1741-2560/12/1/016011 25514320

[B150] Woolley A. J. Desai H. A. Otto K. J. (2013). Chronic intracortical microelectrode arrays induce non-uniform, depth-related tissue responses. J. Neural Eng. 10:026007. 10.1088/1741-2560/10/2/026007 23428842 PMC4096286

[B151] Xie X. Rieth L. Tathireddy P. Solzbacher F. (2011). Long-term in-vivo investigation of parylene-c as encapsulation material for neural interfaces. Procedia Eng. 25, 483–486. 10.1016/j.proeng.2011.12.120

[B152] Yang Z. Zhao Q. Keefer E. Liu W. (2009). Noise characterization, modeling, and reduction for *in vivo* neural recording, in Advances in Neural Information Processing Systems, 2160–2168. Available online at: https://authors.library.caltech.edu/65821/1/3695-noise-characterization-modeling-and-reduction-for-in-vivo-neural-recording.pdf (accessed May 31, 2019).

[B153] Young D. Willett F. Memberg W. D. Murphy B. Walter B. Sweet J.. (2018). Signal processing methods for reducing artifacts in microelectrode brain recordings caused by functional electrical stimulation. J. Neural Eng. 15:026014. 10.1088/1741-2552/aa9ee8 29199642 PMC5818316

[B154] Zhang M. Schwemmer M. A. Ting J. E. Majstorovic C. E. Friedenberg D. A. Bockbrader M. A.. (2018). Extracting wavelet based neural features from human intracortical recordings for neuroprosthetics applications. Bioelectron. Med. 4:11. 10.1186/s42234-018-0011-x 32232087 PMC7098253

